# Evidence update for the treatment of anaphylaxis

**DOI:** 10.1016/j.resuscitation.2021.04.010

**Published:** 2021-06

**Authors:** Amy Dodd, Anna Hughes, Nicholas Sargant, Andrew F. Whyte, Jasmeet Soar, Paul J. Turner

**Affiliations:** a Severn Deanery, UK; b Bristol Royal Hospital for Children, Bristol, UK; c University Hospitals Plymouth NHS Trust, Plymouth, UK; d North Bristol NHS Trust, Bristol, UK; e National Heart and Lung Institute, Imperial College London, UK

**Keywords:** Adrenaline, Anaphylaxis, Antihistamine, Corticosteroids, Resuscitation

## Abstract

The Resuscitation Council UK has updated its Guideline for healthcare providers on the Emergency treatment of anaphylaxis. As part of this process, an evidence review was undertaken by the Guideline Working Group, using an internationally-accepted approach for adoption, adaptation, and de novo guideline development based on the Grading of Recommendations Assessment, Development and Evaluation (GRADE) evidence to decision (EtD) framework, referred to as GRADE-ADOLOPMENT. A number of significant changes have been made, which will be reflected in the updated Guideline. These include: emphasis on repeating intramuscular adrenaline doses after 5 min if symptoms of anaphylaxis do not resolve; corticosteroids (e.g. hydrocortisone) no longer being routinely recommended for the emergency treatment of anaphylaxis; interventions for reactions which are refractory to initial treatment with adrenaline; a recommendation *against* the use of antihistamines for the acute management of anaphylaxis; and guidance relating to the duration of observation following anaphylaxis, and timing of discharge.

## Introduction

The World Allergy Organisation (WAO) defines anaphylaxis as “a serious systemic hypersensitivity reaction that is usually rapid in onset and may cause death. Severe anaphylaxis is characterized by potentially life-threatening compromise in airway, breathing and/or the circulation, and may occur without typical skin features or circulatory shock being present”.^1^ Anaphylaxis thus lies along a spectrum of severity, ranging from mild objective breathing problems (such as mild wheezing) to circulatory “shock” and/or collapse (“anaphylactic shock”). The estimated incidence for anaphylaxis in Europe is 1.5 to 7.9 per 100,000 person-years, with a lifetime prevalence of 1 in 300.^2^ International guidelines concur that the first line treatment of anaphylaxis is intramuscular (IM) adrenaline,^3^ but there is increasing divergence between published guidelines.^4^ This may be due to a lack of high-certainty evidence to support treatment recommendations.^5^ Given the difficulties of undertaking randomised controlled trials in the management of a potentially life-threatening condition, guidelines must therefore be based on the best available research evidence, theory and expert consensus.

**Fig. 1:**
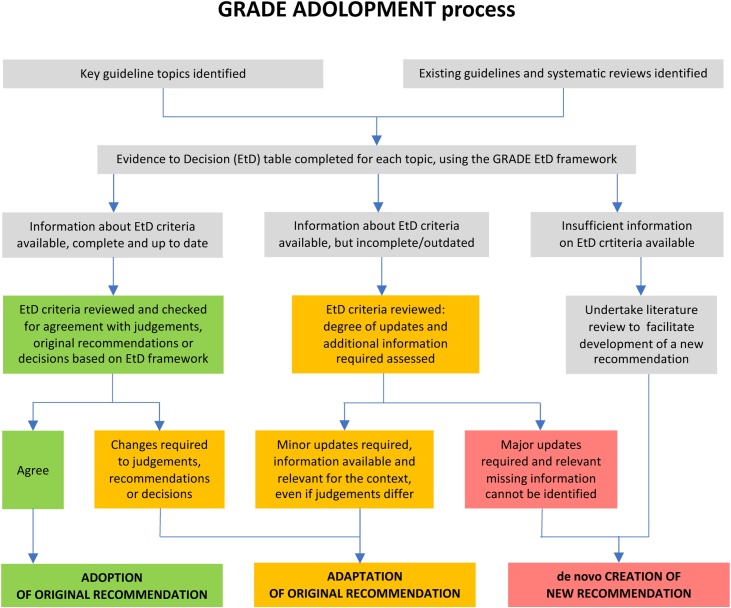
GRADE ADOLOPMENT process.

This evidence review was undertaken by the Anaphylaxis Working Group of the Resuscitation Council UK (RCUK), to support the 2021 update of the RCUK guidelines for the emergency treatment of anaphylaxis. The Working Group used an internationally-accepted approach for adoption, adaptation, and de novo guideline development based on the Grading of Recommendations Assessment, Development and Evaluation (GRADE) evidence to decision (EtD) framework, referred to as GRADE-ADOLOPMENT.^6^ The EtD framework facilitates the use of evidence in a structured and transparent way to inform decisions in the context of clinical and public health recommendations and decisions.^7^The approach is outlined in Fig. 1. In brief, key research questions (see Table 1) were identified from the previous RCUK guideline. The EtD framework for each question/topic, incorporating a review of existing guidelines and published systematic reviews, was independently completed by two assessors. We included international guidelines irrespective of whether they used the GRADE EtD framework (and some guidelines preceded the EtD methodology). The EtD tables were then reviewed by the Working Group, and a consensus reached as to (i) the certainty of the available evidence (Table 2) and (ii) whether this supported the previous recommendation (“adopted”), indicated a need to update the recommendation (“adapted”) or develop an entirely new recommendation. The strength for each recommendation was assigned as either strong or weak (see Table 3).^8^ Reasons for a weak recommendation include: the absence of high-certainty evidence; imprecision in outcome estimates; variability in the values and preferences of individuals regarding the outcomes of interventions; small benefits; applicability in all settings versus specific settings; and benefits that may not be worth the costs (including the costs of implementing the recommendation). These criteria are summarised in Table S1, supplementary material. Finally, recommendations and their evidence base were reviewed by a Consultation Panel (see acknowledgements) prior to a public consultation (via the Resuscitation Council UK website, between 23 December 2020 and 24 February 2021, resulting in 130 submissions) and finalisation by the working group.

**Table 1:** Identified research questions for evaluation.

RCUK 2008 recommendation	Research question for review
Adrenaline is the most important drug for the treatment of an anaphylactic reaction. The intramuscular (IM) route for adrenaline is the route of choice for most healthcare providers.	Is adrenaline effective for the treatment of anaphylaxis?
	What is the optimal timing of adrenaline in the treatment of anaphylaxis?
	What is the optimal route of adrenaline to treat anaphylaxis?
Adrenaline IM dose – Adults 0.5 mg IM – Children: the scientific basis for the recommended doses is weak.	What is the optimal dose of intramuscular adrenaline in the treatment of anaphylaxis?
Repeat the IM adrenaline dose if there is no improvement in the patient's condition. Further doses can be given at about 5-min intervals according to the patient's response.	Is adrenaline effective in the treatment of anaphylaxis reactions refractory to initial treatment with adrenaline?
Large volumes of fluid may leak from the patient's circulation during an anaphylactic reaction… Give a rapid IV fluid challenge and monitor the response; give further doses as necessary.	Are intravenous fluids effective as an adjuvant treatment for anaphylaxis?
Antihistamines are a second line treatment for an anaphylactic reaction. The evidence to support their use is weak, but there are logical reasons for them. Before discharge from hospital all patients must be… considered for anti-histamines and oral steroid therapy for up to 3 days	Are antihistamines effective in the treatment of anaphylaxis?
Corticosteroids may help prevent or shorten protracted reactions. Before discharge from hospital all patients must be… considered for anti-histamines and oral steroid therapy for up to 3 days	Are corticosteroids effective in the treatment of anaphylaxis?
Consider further bronchodilator therapy with salbutamol (inhaled or IV), ipratropium (inhaled), aminophylline (IV) or magnesium (IV).	Are inhaled beta-2 agonists effective in the treatment of anaphylaxis?
Patients should be… observed for at least 6 h in a clinical area with facilities for treating life-threatening ABC problems	How long should patients be observed in hospital following anaphylaxis?

**Table 2:** Certainty of evidence.^6^

Certainty of evidence	Explanation
High	We are very confident that the true effect lies close to that of the estimate of the effect
Moderate	We are moderately confident in the effect estimate: The true effect is likely to be close to the estimate of the effect, but there is a possibility that it is substantially different
Low	Our confidence in the effect estimate is limited: the true effect may be substantially different from the estimate of the effect
Very low	We have very little confidence in the effect estimate: the true effect is likely to be substantially different from the estimate of effect

**Table 3:** Interpretation of strong and weak recommendations.^8^

Implications	Strong recommendation	Weak recommendation
For patients	Most individuals in this situation would want the recommended course of action and only a small proportion would not. Formal decision aids are not likely to be needed to help individuals make decisions consistent with their values and preferences.	The majority of individuals in this situation would want the suggested course of action, but many would not.
For clinicians	Most individuals should receive the intervention. Adherence to this recommendation according to the guideline could be used as a quality criterion or performance indicator.	Recognize that different choices will be appropriate for individual patients and that you must help each patient arrive at a management decision consistent with his or her values and preferences. Decision aids may be useful helping individuals making decisions consistent with their values and preferences.
For policy makers	The recommendation can be adapted as policy in most situations.	Policymaking will require substantial debate and involvement of various stakeholders.

The guidelines reviewed were those from: British Society for Allergy & Clinical Immunology (BSACI)^9^, ^10^; National Institute for Health and Care Excellence (NICE)^11^; European Academy of Allergy and Clinical Immunology (EAACI)^12^, ^13^; Australasian Society of Clinical Immunology and Allergy (ASCIA)^14^; Joint Task Force on Practice Parameters (JTFPP) of the American Academy of Allergy, Asthma & Immunology (AAAAI) and the American College of Allergy, Asthma and Immunology (ACAAI)^15^, ^16^; Canadian Society of Allergy and Clinical Immunology (CSACI)^17^; World Allergy Organisation (WAO).^1^, ^3^, ^18^, ^19^, ^20^, ^21^, ^22^ The EAACI 2021 updated guideline and JTFPP 2020 documents followed the GRADE EtD framework. Systematic reviews of anaphylaxis treatment (including both randomised controlled trials and observational studies) published in the last 10 years were identified by searching PubMed and Cochrane Database of Systematic Reviews.

### Is adrenaline effective for the treatment of anaphylaxis?

#### Recommendation

We recommend adrenaline as the first line treatment for anaphylaxis (strong recommendation, moderate certainty evidence)

*(adopted from RCUK 2008 and EAACI 2014 guidelines)*

#### Rationale

International guidelines agree that adrenaline (epinephrine) is first line treatment for anaphylaxis. However, supporting evidence is limited to observational studies (case series and fatality registers) in humans, animal models, epidemiological studies, and pharmacokinetic studies in patients who might be at risk for anaphylaxis but not experiencing allergic symptoms at the time of study. The EAACI 2014 guideline concluded “there is some evidence to support the use of adrenaline for the emergency management of anaphylaxis”,^12^ while the WAO 2011 Guideline noted that “the evidence base for prompt epinephrine injection in the initial treatment of anaphylaxis is stronger than the evidence base for the use of antihistamines and glucocorticoids in anaphylaxis”.^18^ A systematic review by EAACI in 2020 only identified observational studies examining the efficacy of adrenaline and noted a high risk of bias; no eligible studies compared adrenaline with no adrenaline for critical outcomes such as mortality, or most other outcomes.^23^

There is little doubt that sufficient adrenaline results in symptom resolution, and that delayed administration is associated with protracted reactions, hypotension and fatal outcomes.^23^, ^24^ Fatal outcomes due to anaphylaxis are rare,^25^, ^26^ and around 80% of reactions resolve without (or despite no treatment with) adrenaline.^27^, ^28^ However, severe reactions cannot be predicted,^1^ thus all anaphylaxis reactions must be treated as potentially life-threatening. At least one-third of deaths due to food-induced anaphylaxis in the UK occur despite timely administration of adrenaline;^29^ observational studies^30^ and data from animal models^31^ indicate that this is likely due to severe reactions requiring more than one or two doses of IM adrenaline. Around 10% of anaphylaxis events demonstrate a suboptimal response to a single dose of adrenaline; most will respond to one or two further doses.^32^

Overall, the evidence for adrenaline to treat anaphylaxis was graded as moderate certainty (Table 2) – while confidence in the effect estimate is limited, data from a systematic review and meta-analysis (including 36,557 anaphylaxis events) indicates that only 2.2% (95% CI 1.1–4.1%) of reactions fail to respond to two doses of adrenaline.^32^ It was deemed very unlikely that the true effect would be substantially different from this estimate, thus under the EtD framework (Table 2) the certainty was assigned as moderate. The strong recommendation for adrenaline is based on the working group placing a high value on evidence suggesting that adrenaline is the most appropriate treatment to reduce morbidity, recommendations for its use in existing anaphylaxis guidelines, and feedback from the public consultation.

Anaphylaxis may resolve but then exhibit a recrudescence several hours later in the absence of further exposure to an allergen (biphasic reaction). A systematic review and meta-analysis of 27 studies (2758 patients, 5% rate of biphasic reactions) reported no impact of adrenaline treatment on the occurrence of biphasic reactions (pooled OR 0.91, 95% CI 0.6–1.4).^33^ This is consistent with data from the European Anaphylaxis Registry (7328 patients, 5% rate of biphasic reactions; OR 0.91, 95% CI 0.71–1.16).^34^ The EAACI 2020 systematic review reported two relevant case-control studies, but could not comment on whether adrenaline prevents biphasic anaphylactic reactions because the certainty of evidence was very low.^23^

### What is the optimal timing of adrenaline in the treatment of anaphylaxis?

#### Recommendation

Adrenaline should be administered early once symptoms of anaphylaxis have been recognized or suspected (weak recommendation, very low certainty evidence).

*(adopted from RCUK 2008 and EAACI 2014 guidelines)*

#### Rationale

There is a lack of high-certainty evidence to differentiate the effect of early versus delayed administration of adrenaline on clinical outcomes.^23^ Case series (including reports of fatal anaphylaxis) suggest that early adrenaline administration for out-of-hospital anaphylaxis is associated with improved outcomes.^12^ There is no evidence that pre-emptive use of adrenaline to treat mild, non-anaphylaxis reactions prevents progression to anaphylaxis.^35^ However, despite the lack of evidence to inform the optimal timing of administration,^23^ it seems reasonable to recommend adrenaline is given as soon as features of anaphylaxis are apparent; this is the consensus reflected in international guidelines.

With respect to biphasic reactions, the 2020 JTFPP identified eight retrospective case series, three of which found that delayed administration was associated with a higher rate of biphasic reaction.^16^ A prospective cohort study of 430 anaphylaxis reactions found that delayed administration of adrenaline (more than 30 min after onset of symptoms) was associated with a higher rate of biphasic reaction (OR 3.39, 95% CI 1.13–10.18).^36^ The 2020 JTFPP concluded that “there does appear to be a trend to lower rates of biphasic reactions with earlier epinephrine administration following development of anaphylaxis”.^16^

### What is the optimal route of adrenaline to treat anaphylaxis?

#### Updated recommendations

1. The intramuscular (IM) route is recommended for initial adrenaline treatment for anaphylaxis (strong recommendation, very low certainty evidence).
2. The intravenous (IV) route is not recommended for initial management of anaphylaxis, except in the perioperative setting (as an alternative to IM adrenaline) by those skilled and experienced in its use (good practice statement).
  • In such circumstances, adrenaline should preferably be administered as an IV infusion and not as a bolus dose (weak recommendation, very low certainty evidence).
3. Titrate the administration of adrenaline (by any route) against clinical response (strong recommendation, very low certainty evidence).

*(adapted from RCUK 2008 and EAACI 2014 guidelines, with greater emphasis on IM route and where needed, use of IV adrenaline infusion rather than IV bolus therapy)*

#### Rationale

There are no trials comparing different routes of adrenaline administration in patients during anaphylaxis. IM adrenaline is recommended over other routes of administration for initial treatment of anaphylaxis, due to a favourable adverse event profile (including in those with cardiovascular co-morbidities).^1^, ^12^ The subcutaneous route is not recommended, on the basis of (low certainty) evidence that higher plasma adrenaline levels are achieved by the IM route;^37^ the available data relates to pharmacokinetic studies undertaken in patients outside the context of an allergic reaction and “may be confounded by using different injection sites (thigh versus arm), in addition to different depth of injection”.^23^ Comparing the IM to IV route, the EAACI 2020 systematic review identified a single case series (children and adults) which found that “IV bolus administration was associated with a 13% increase in the incidence of adrenaline overdose and an 8% increase in the incidence of cardiovascular events compared with IM administration”.^38^ Excessive doses of adrenaline, particularly by the IV route, can cause tachyarrhythmias, severe hypertension, myocardial infarction and stroke. Fatalities have occurred in the UK due to the inappropriate use of intravenous adrenaline to treat allergic (but non-anaphylaxis) reactions.^39^ Both IM and IV routes are recommended for treating perioperative anaphylaxis by experienced anaesthetists,^40^, ^41^ although international guidelines recommend IM adrenaline for first-line treatment of anaphylaxis in all settings. If cardiac arrest is imminent or has already occurred, an intravenous (or interosseous) bolus dose of adrenaline is indicated.^4^

Although the evidence was assessed as being of low certainty, the working group agreed with the evaluation in other guidelines that “given the totality of the evidence and clinical experience over many decades… a strong recommendation for the use of intramuscular adrenaline was appropriate”.^13^ A strong recommendation for the IM route was deemed justified, as the working group placed a high value on the relative ease and safety of IM adrenaline administration by a wide variety of healthcare staff, and the current acceptance of the IM route in both clinical and non-clinical settings (including by patients for self-administration using an autoinjector device). Despite the limited evidence, we have made a strong recommendation for titrating the dose of adrenaline (as an intravenous infusion) against the clinical response, since this is routine in clinical practice to mitigate against the side effects of excessive adrenaline administration.

### What is the optimal dose of intramuscular adrenaline in the treatment of anaphylaxis?

#### Recommendation

Intramuscular adrenaline should be administered at the doses listed in Table 4: (strong recommendation, low certainty evidence)

**Table 4:** Recommended doses of IM adrenaline.

*Adrenaline IM dose – adults*	
500 micrograms (0.5 mg) IM (0.5 mL of 1 mg/ml [1:1000] adrenaline)	

*Adrenaline IM dose – children*	
>12 years	500 micrograms IM (0.5 mL) i.e. same as adult dose 300 micrograms (0.3 mL) if child is small or prepubertal
6–12 years	300 micrograms IM (0.3 mL)
6 months–6 years	150 micrograms IM (0.15 mL)
<6 months	100–150 micrograms IM (0.1–0.15 mL)

The equivalent volume of 1 mg/ml [1:1000] adrenaline is shown in brackets.

*(adopted from RCUK 2008 and EAACI 2014 guidelines)*

#### Rationale

The safety and efficacy of the dosing regimen (Table 4) has been established in clinical practice for over 20 years. In children, a dose of 0.01 mg/kg (max 500 microgram) titrated to clinical response is recommended in international guidelines. Many guidelines (including those from EAACI, WAO and RCUK) simplify the dosing regimen to age categories, based on what is considered to be safe and practical to draw up and inject in an emergency.^42^ This pragmatic approach (which matches the licensed doses used for auto-injectors) seems to be effective and safe. Four small crossover RCTs have been published which compare different doses of IM adrenaline: one in children (weight 15–30 kg) comparing 150/300 micrograms;^43^ and three comparing 300/500 micrograms in teenagers^44^ or adults.^45^, ^46^ In all four studies, the higher dose had a more favourable absorption profile, however how this impacts on clinical response in patients with anaphylaxis is unknown. While the certainty of evidence with respect to dose is low, the working group concluded that a strong recommendation was appropriate given these doses have been widely used globally for many decades. In addition, we did not identify any new evidence to challenge current dosing recommendations.

In terms of the practicalities of IM administration, the EAACI 2020 systematic review identified one study in which untrained caregivers were more able to give adrenaline using a prefilled syringe correctly, than when using an adrenaline auto-injector (AAI) (OR 4.07, 95%CI 1.29–12.86; low certainty).^47^ A study with radiologists found that using an AAI reduced the time to administration by an average of 70 s compared to drawing up manually from an ampoule, and resulted in fewer administration errors.^48^ Most AAIs deliver a maximum of 300 micrograms epinephrine, while the appropriate dose in teenagers and adults is 500 micrograms. Coronial inquests have identified that the use of AAIs for anaphylaxis can therefore result in substantial underdosing, which may contribute to fatal outcomes.^49^, ^50^ A single-blinded, cross-over RCT in 12 food-allergic teenagers reported that a 500 microgram dose (given by AAI) had a more favourable pharmacokinetic and pharmacodynamic profile compared to 300 micrograms, without causing a higher rate of systemic adverse events.^44^ Therefore, while some settings may prefer to use an AAI to administer an initial dose of adrenaline (for speed and ease), further doses should be given by needle/syringe in order to deliver an optimal dose.

### Are additional doses of adrenaline effective in the treatment of anaphylaxis reactions refractory to initial treatment with adrenaline?

#### Updated recommendations

1. Subsequent doses of adrenaline should be given every 5 min, titrated to clinical response, in patients whose symptoms are refractory to initial treatment (weak recommendation, very low certainty evidence).
2. Where respiratory and/or cardiovascular features of anaphylaxis persist despite 2 appropriate doses of adrenaline (administered by IM or IV route), seek urgent expert help (e.g. from experienced critical care clinicians) to establish an intravenous adrenaline infusion to treat refractory anaphylaxis (strong recommendation, low certainty evidence).
3. Low dose intravenous adrenaline infusions appear to be effective and safe to treat refractory anaphylaxis (weak recommendation, very low certainty evidence).

*(adapted from RCUK 2008, EAACI 2014 and ASCIA 2020 guidelines, with greater emphasis on early recognition of refractory reactions and further adrenaline treatment, preferably using a low dose IV adrenaline infusion)*

#### Rationale

Around 10% of anaphylaxis reactions (predominantly community reactions to food allergens) have a suboptimal response to a single dose of IM adrenaline, but 98% will respond to 1 or 2 further doses.^32^ While effective for respiratory symptoms, a single dose of IM adrenaline has a limited effect on reversing the decrease in stroke volume seen during peanut-induced anaphylaxis.^51^ Case series of refractory anaphylaxis^30^, ^52^ and evidence from animal models^31^, ^53^ indicate that a poor response to adrenaline is likely due to *insufficient* adrenaline delivery (a combination of both inadequate dosing with adrenaline, and insufficient circulatory capacity to ensure adequate dose-distribution).

The absorption of adrenaline following IM injection follows a biphasic profile, with the initial peak occurring within 5–10 min.^37^ International guidelines agree that IM adrenaline should be repeated every 5–15 min where features of anaphylaxis persist;^12^, ^13^, ^14^, ^15^, ^16^, ^17^, ^18^, ^19^, ^20^, ^21^ the rationale for waiting longer than 5 min where symptoms have failed to respond to adrenaline is unclear. In a canine model of anaphylactic shock, a low dose intravenous adrenaline infusion resulted in a far better haemodynamic profile compared to IM or IV bolus treatment.^31^ Low dose adrenaline infusions are efficacious in case series of human anaphylaxis,^30^, ^54^ and are included as the treatment of choice for refractory anaphylaxis in national guidelines in Australia^14^ and Spain.^55^ Complications due to adrenaline occur regardless of route but are more common after IV administration, particularly with “overly rapid intravenous infusion, bolus administration, and dosing error”, for example using 1 mg/ml (1:1000) solution (appropriate for IM injection) instead of more dilute solutions e.g. 0.1 mg/ml (1:10,000) for intravenous injections.^18^ These concerns need to be balanced against the risk of death due to refractory anaphylaxis. Reassuringly, appropriate use of low dose intravenous adrenaline infusions appears to both efficacious and safe.^30^, ^54^ At least 98% of reactions reported in the literature respond to a maximum of 3 doses of IM adrenaline.^32^ The working group therefore suggests that following a suboptimal response to 2 doses of adrenaline, expert input is urgently sought to establish a low dose IV adrenaline infusion to provide further vasopressor support (on the basis that this will take at least 5 min to set-up, during which a third bolus dose of IM/IV adrenaline should be administered). Given the potential risks of intravenous adrenaline infusion without the necessary expertise and support, and evidence supporting the use of intravenous adrenaline infusions for refractory reactions, we make a *strong* recommendation that urgent expert support is obtained to establish an intravenous adrenaline infusion to treat refractory anaphylaxis.

With respect to second-line vasopressors, “no clear superiority of dopamine, dobutamine, norepinephrine, phenylephrine, or vasopressin (either added to [adrenaline] alone, or compared with one another), has been demonstrated in clinical trials”.^18^ The ASCIA 2020 Guideline recommends consideration of other vasopressors or inotropes only if an IV adrenaline infusion is ineffective.^14^ Animal models suggest that early treatment with adrenaline followed by continuous adrenaline or vasopressin infusion is superior to vasopressin alone,^53^, ^56^ thus confirming that adrenaline must be considered the first-line intervention to treat anaphylactic shock.

### Are intravenous fluids effective as an adjuvant treatment for anaphylaxis?

#### Updated recommendations

1. In the presence of anaphylaxis with haemodynamic compromise, intravenous (IV) crystalloid fluids should be infused (weak recommendation, very low certainty evidence).
2. For anaphylaxis refractory to initial treatment with adrenaline, an IV fluid bolus (crystalloid) is recommended as an adjunct to improve drug distribution (weak recommendation, very low certainty evidence).

*(adapted from RCUK 2008, EAACI 2014 and ASCIA 2020 guidelines, with addition of fluid bolus to treat refractory reactions even in the absence of obvious haemodynamic compromise)*

#### Rationale

Evidence from observational studies and animal models strongly suggests that anaphylactic shock occurs as a consequence of a profound reduction in venous tone and fluid extravasation. Allergic mediators can also impair cardiac function. This results in a mix of hypovolaemic, distributive and possibly cardiogenic shock, which combine to reduce venous return.^57^ Guidelines recommend (on the basis of expert consensus) that intravenous fluids are administered to patients with cardiovascular instability, as adrenaline may not be effective without restoring the circulatory volume.^1^, ^12^, ^14^

In peanut-allergic adults, stroke volume was reduced during even mild (non-anaphylaxis) reactions (presumably due to a drop in venous return), although cardiac output was in general maintained due to a compensatory tachycardia.^58^ A related study in the same cohort found that a single dose of IM adrenaline had limited effect in restoring stroke volume.^51^ A 500–1000 mL crystalloid infusion had greater effect in restoring venous return compared to a single dose of IM adrenaline.^58^ It therefore seems prudent to administer an IV fluid bolus in all cases of anaphylaxis refractory to initial treatment, irrespective of whether there is evidence of haemodynamic compromise. The restoration of circulating volume may aid adrenaline delivery and hasten symptom resolution. A single bolus of IV crystalloid is unlikely to cause overload in the context of anaphylactic shock or refractory anaphylaxis, and judicious use of IV fluids, titrated to clinical response, is potentially lifesaving.

### Are antihistamines effective in the treatment of anaphylaxis?

#### Updated recommendations:

1. We suggest that antihistamines are not used as part of the initial emergency treatment for anaphylaxis (weak recommendation, low certainty evidence)
  - antihistamines have no role in treating respiratory or cardiovascular symptoms of anaphylaxis
  2. We suggest antihistamines are used to treat skin symptoms which often occur as part of allergic reactions including anaphylaxis (weak recommendation, very low certainty evidence)
  - their use must not delay management of respiratory or cardiovascular symptoms of anaphylaxis (using adrenaline and IV fluids).

*(adapted from RCUK 2008, WAO 2011/2020, EAACI 2014 and ASCIA 2020 guidelines, with greater emphasis on the risks of antihistamines delaying timely and appropriate use of adrenaline to treat anaphylaxis)*

#### Rationale

There is no RCT or quasi-RCT evidence to support the use of antihistamines in treating anaphylaxis.^1^, ^12^, ^21^ Antihistamines do not lead to resolution of respiratory or cardiovascular symptoms of anaphylaxis, or improve survival.^16^, ^59^, ^60^ H1-antihistamines cause sedation which can confound symptoms of anaphylaxis,^14^ and if given by rapid intravenous bolus may precipitate hypotension.^1^, ^12^, ^61^ Recent guidelines relegate antihistamines to a second or third-line intervention; most express a concern that their use can delay the administration of both initial and subsequent doses of adrenaline.^1^, ^12^, ^14^, ^16^ This is based on a large number of datasets which report that the majority of patients presenting with anaphylaxis to Emergency Departments are treated with antihistamines, yet only a minority receive adrenaline – despite an increasing emphasis on adrenaline as the first-line intervention in international guidelines.^62^, ^63^, ^64^, ^65^, ^66^, ^67^, ^68^ In a large, national prospective registry (Cross-Canada Anaphylaxis Registry, C-CARE), 3498 cases of anaphylaxis were enrolled over a 6 year period; prehospital antihistamine use was associated with a lower rate of administration of >1 adrenaline dose (adjusted OR 0.61; 95% CI 0.44–0.85), but not other outcomes (hospitalisation/intensive care, intravenous fluids). Moreover, this finding was not robust at sensitivity analyses: excluding less severe reactions, prehospital antihistamine did not affect outcomes; unfortunately, the authors did not assess the impact on >2 doses of adrenaline being given.^68^ An association between pre-hospital antihistamine use and delayed presentation to healthcare facilities has been reported, resulting in delays in adrenaline administration and increased morbidity.^69^ Antihistamines do not reduce the occurrence of biphasic reactions.^16^, ^33^ An analysis of 9171 anaphylaxis episodes reported to the European Anaphylaxis Register found that antihistamine treatment was significantly associated with the occurrence of biphasic reactions (OR 1.52, 95% CI 1.14–2.02);^34^ this may be due to antihistamine use delaying adrenaline administration. We therefore recommend against antihistamines for the acute management of anaphylaxis (weak recommendation); this in consistent with the ASCIA 2020 Guideline.^14^

Oral H1-antihistamines relieve the cutaneous symptoms of anaphylaxis; combined H1- and H2-antihistamines may be more effective than H1-antihistamines alone, although data are limited.^12^ However, cutaneous symptoms are not life-threatening and also respond to adrenaline (although the effect may not be long-lasting). The ASCIA 2020 guideline cautions against the use of sedating antihistamines as “side effects (drowsiness or lethargy) may mimic some signs of anaphylaxis”.^14^ Antihistamines may be helpful in treating cutaneous symptoms that persist following resolution of anaphylaxis symptoms, but are not recommended until the acute reaction has been successfully treated with more appropriate interventions.^1^, ^12^, ^13^, ^14^, ^15^, ^16^, ^17^ A non-sedating oral antihistamine is preferred, to avoid confounding due to the risk of sedation which can indicate reaction progression.

### Are corticosteroids effective in the treatment of anaphylaxis?

#### Updated recommendations

1. We suggest *against* the routine use of corticosteroids to treat anaphylaxis (weak recommendation, very low certainty evidence).
2. We suggest corticosteroids may be used as a third line intervention to treat underlying asthma or shock (weak recommendation, very low certainty evidence)

*(adapted from RCUK 2008 and JTFPP 2020 guidelines, in view of new data which casts further doubt on the efficacy of steroids to prevent biphasic reactions and possibility of harm (increased need for hospitalisation) in at least one study)*

#### Rationale

The primary action of corticosteroids is the downregulation of the late (rather than early) phase inflammatory response. Given the (slow) absorption kinetics of corticosteroids and their mechanism of action (i.e. through an inhibitory effect on proinflammatory transcription factors such as nuclear factor-κB), it is theoretically unlikely that corticosteroids are of benefit in the acute treatment of anaphylaxis;^16^, ^68^ the rationale for use is therefore to prevent biphasic reactions. However, a 2012 Cochrane systematic review concluded “clinicians should be aware of the lack of a strong evidence base for the use of a glucocorticoid for anaphylaxis”.^70^ Subsequent systematic reviews have confirmed the absence of evidence that corticosteroids reduce reaction severity or prevent biphasic reactions.^16^, ^71^

As with antihistamines, corticosteroids are administered far more frequently than adrenaline for the acute treatment of anaphylaxis,^62^, ^63^, ^64^, ^65^, ^66^, ^67^, ^68^, ^70^ implying that their use may distract from the need to administer adrenaline. However, of greater concern is emerging evidence that routine use of corticosteroids for anaphylaxis may be harmful, and associated with increased morbidity even after correcting for confounding by indication.^68^, ^72^ In the Canadian C-CARE registry, hospitalisation and/or admission to intensive care was associated with prehospital treatment with corticosteroids (OR 2.84; 95% CI 1.55–6.97, adjusted for reaction severity and treatments administered).^68^ It is unclear why steroids might increase morbidity: the association was present even after adjusting for prehospital adrenaline.

We therefore recommend against the *routine* use of corticosteroids to treat anaphylaxis (weak recommendation). Corticosteroids may be of benefit in the following specific scenarios: refractory anaphylaxis (defined as anaphylaxis requiring ongoing treatment despite two appropriate doses of IM adrenaline) and anaphylaxis occurring in the context of poorly-controlled asthma. With the absence of evidence in such cases and the possibility of a different risk:benefit ratio, it is reasonable to include corticosteroids as part of the management for refractory anaphylaxis, but only as an adjunct and not in preference to adrenaline or other inotropes/vasopressor agents.

### Are inhaled beta-2 agonists effective in the treatment of anaphylaxis?

#### Updated recommendation

1. Beta-2 agonists (such as salbutamol) may be useful as an adjunct treatment for lower respiratory symptoms caused by anaphylaxis, following initial treatment with IM adrenaline (weak recommendation, very low certainty evidence).
2. In the presence of persisting respiratory symptoms in anaphylaxis, beta-2 agonists (whether inhaled or parenteral) should not be used as an alternative to further parenteral treatment with adrenaline (strong recommendation, very low certainty evidence).

*(adapted from RCUK 2008, WAO 2011/2020, EAACI 2014 and ASCIA 2020 guidelines, with greater emphasis on using bronchodilators as an adjunct rather than a replacement for adrenaline)*

#### Rationale

Beta-2 agonists are widely used in clinical practice and feature in most guidelines as a second-line treatment option for anaphylaxis. There is limited evidence to support the use of inhaled beta-2 agonists in the emergency treatment of anaphylaxis and evidence is extrapolated from their use to treat acute asthma.^1^, ^12^, ^18^ International guidelines agree that bronchodilators may be helpful for persisting wheeze, but caution that they do not prevent or relieve upper airway obstruction, hypotension or shock, and should therefore be used as adjunct treatments.^1^, ^12^, ^14^, ^17^

In patients with mild to moderate respiratory symptoms, beta-2 agonists can be administered by repeated activations of a Metered Dose Inhaler (MDI) via an appropriate large volume spacer where the patient does not require supplementary oxygen. There are insufficient data to make a recommendation over the use of MDIs with spacers in acute severe or life-threatening respiratory symptoms; in these patients, beta-2 agonists should be administered by oxygen-driven nebuliser. There are anecdotal reports of anaphylaxis initially misdiagnosed as severe asthma, which did not respond to parenteral bronchodilator therapy but did respond to adrenaline.^73^, ^74^ For this reason, parenteral beta-2 agonists (such as intravenous salbutamol) must not be used in preference to adrenaline for acute anaphylaxis. This recommendation is made on the basis of adrenaline (including further doses) being established as the first-line treatment of anaphylaxis.

### How long should patients be observed in hospital following anaphylaxis?

#### Updated recommendation

We suggest a risk-stratified approach to the discharge of patients following anaphylaxis (Table 5) (weak recommendation, very low certainty evidence).

**Table 5:** Suggested observation times following anaphylaxis.

Consider fast-track discharge (after 2 h observation from resolution of anaphylaxis) if:	Minimum 6 h observation after resolution of symptoms recommended if:	Observation for at least 12 h following resolution of symptoms if any one of the following:
• Good response (within 5–10 min) to a single dose of adrenaline given within 30 min of onset of reaction; AND • Complete resolution of symptoms AND • The patient already has unused adrenaline auto-injectors (AAI) and has been trained how to use them. AND • There is adequate supervision following discharge	• 2 doses of IM adrenaline needed to treat reaction^a^ OR • Previous biphasic reaction	• Severe reaction requiring >2 doses of adrenaline. • Patient has severe asthma or reaction involved severe respiratory compromise. • Possibility of continuing absorption of allergen e.g. slow release medicines. • Patient presents late at night, or may not be able to respond to any deterioration. • Patients in areas where access to emergency care is difficult.

a It may be reasonable for some patients to be discharged after 2 h despite needing no more than 2 doses of IM adrenaline e.g. following a supervised allergy challenge in a specialist setting.

*(adapted from RCUK 2008, NICE 2011 and JTFPP 2020 guidelines)*

#### Rationale

The recurrence of anaphylaxis symptoms following initial resolution may be a “biphasic” reaction but can also represent (and be difficult to distinguish from) protracted anaphylaxis with a transient response to adrenaline, or in the case of food-induced reactions, further allergen absorption from the gastrointestinal tract.^75^ Historical guidelines have suggested a rate of up to 20% for biphasic reactions, however a recent meta-analysis reported a pooled rate of 4.6% (95% CI 4.0–5.3).^33^ A rate of 4.7% has been reported in the European Anaphylaxis Registry.^34^ In a prospective case series of anaphylaxis presenting to Emergency Departments, delayed deteriorations were noted in 17% (55/315) of reactions, of which 29 (9.2%) required treatment with adrenaline.^76^

Contradictory ranges for the onset of biphasic symptoms are reported in the literature. The WAO 2011 guideline states that symptoms can recur within 1–72 h (usually within 8–10 h).^18^ Median times reported in the literature range from 1.7 (Interquartile range 0.7–4.3) hours^76^ to 11 h i.e. 50% of biphasic reactions began more than 11 h after initial symptoms.^33^ In the European Anaphylaxis Registry, one third of biphasic reactions occurred more than 12 h after initial symptoms.^34^

The optimal duration of observation following anaphylaxis is unknown. The previous RCUK guideline recommended patients should be observed for at least 6 h,^5^ on the basis of data from the UK Fatal Anaphylaxis Register which found that in cases reported to 2000, death never occurred more than 6 h after contact with the trigger.^77^ However, in an updated analysis in 2014, 2.5% of fatalities happened > 6 h after allergen exposure.^29^ In 2011, NICE concluded there was “no evidence on the effectiveness of observing people… or how long people should be observed after a suspected anaphylactic reaction”, but in line with RCUK, recommended 6–12 h observation from the onset of symptoms.^11^ The published literature clearly indicates that this strategy will miss over 50% of biphasic reactions.^33^, ^34^, ^76^ NICE recommends that patients under 16 years should be admitted to hospital under a paediatric team to ensure that “children and their parents or carers… receive the appropriate care (for example, paediatric assessment, counselling, education) following emergency treatment.” However, NICE acknowledges that “shorter observation periods could be warranted in those who seek and respond quickly to treatment,” particularly in those with a prior diagnosis who already have a management plan and appropriate rescue medication including AAIs.^11^

The 2020 JTFPP recommends extended observation for patients with severe initial symptoms of anaphylaxis,^16^ based on a meta-analysis which found biphasic anaphylaxis was associated with a more severe initial presentation (OR 2.11, 95% CI 1.23–3.61) or administration of > 1 dose of adrenaline (OR 4.82, 95% CI 2.70–8.58). The JTFPP otherwise suggests that 1 h observation may be reasonable for low-risk patients with resolved non-severe anaphylaxis; this is supported by a 2019 meta-analysis which reported that 1 h observation would capture 95.0% (95%CI 99.0–97.3%) of biphasic reactions.^78^ Extending this interval would only impact slightly on the rate of biphasic reactions “captured”: 96.5% (95%CI 93.4–98.2%) for 4 h, 97.3% (95%CI 95.0–98.5%) for 6 h and 98.2% (95%CI 96.7–99.1%) for 12 h observation. Prolonged observation is inconvenient for many patients (and their carers), and is not cost-effective for patients at low risk of biphasic reactions.^79^

After considering the available evidence, the working group was concerned that the previous RCUK recommendation might offer false reassurance in terms of mitigating against the risk of biphasic reaction. To balance the risks and benefits involved, we instead propose a pragmatic, risk-stratified and individualised approach to determining the length of observation following anaphylaxis (Table 5).

## Discussion

In general, the certainty of evidence underpinning anaphylaxis management is low or very low. The GRADE-ADOLOPMENT process provides a robust and transparent mechanism to assess the current evidence for treatment of anaphylaxis, to inform the 2021 RCUK Anaphylaxis Guideline update. A strength of this approach is that it should reduce discordance between different guidelines, and highlight the reasons for any discrepancies. Through a public consultation, we were able to include responses from key stakeholders, ensuring that our recommendations considered the values and preferences of clinicians, patients and carers. We have previously commented “anaphylaxis is anaphylaxis, irrespective of where it occurs: it does not vary in presentation or response to treatment depending on country or region.” As a community, we need to “achieve an international consensus on what we do know, and transparency over those areas for which (at best) there is limited evidence and at worst, emerging data that such interventions may do harm.”^80^ We hope this evidence review serves as an initial step in this process.

## Conclusion

We used the GRADE-ADOLOPMENT process to evaluate current evidence for the emergency treatment of anaphylaxis, incorporating a public consultation, to inform the updated 2021 Resuscitation Council UK Anaphylaxis Guideline.

## Conflicts of interest

J. Soar is joint-chair of the Anaphylaxis Working group of the UK Resuscitation Council, Editor of Resuscitation and receives payment from the publisher Elsevier. P.J. Turner is supported by a UK Medical Research Council Clinician Scientist award (reference MR/K010468/1) and reports grants from UK Medical Research Council, NIHR/Imperial Biomedical Research Centre, UK Food Standards Agency, End Allergies Together, and Jon Moulton Charity Trust; personal fees and nonfinancial support from Aimmune Therapeutics, DBV Technologies, and Allergenis; personal fees and other fees from ILSI Europe and UK Food Standards Agency, outside the submitted work; and is current Chairperson of the WAO Anaphylaxis Committee, Chairperson of the Paediatric Allergy Group of the British Society for Allergy and Clinical Immunology, and joint-chair of the Anaphylaxis Working group of the UK Resuscitation Council. A.F. Whyte is current Chairperson of the Adult Allergy Group of the British Society for Allergy and Clinical Immunology. The rest of the authors declare that they have no relevant conflicts of interest.

## CRediT authorship contribution statement

Amy Dodd: Methodology, Analysis, Writing - original draft, Writing - review & editing.

Anna Hughes: Methodology, Analysis, Writing - original draft, Writing - review & editing. Nicholas Sargant: Methodology, Analysis, Writing - review & editing. Andrew F Whyte: Methodology, Analysis, Writing - review & editing. Jasmeet Soar: Conceptualization, Methodology, Analysis, Writing - review & editing, Project administration. Paul J. Turner: Conceptualization, Methodology, Analysis, Writing - original draft, Writing - review & editing, Project administration.

## Declaration of Competing Interest

The authors report no declarations of interest.
