# Supplementary material for: Evidence update for the treatment of anaphylaxis
Source: Resuscitation. 2021 Jun;163:86–96. doi: 10.1016/j.resuscitation.2021.04.010 (PMC8139870; doi:10.1016/j.resuscitation.2021.04.010)
Supplement: Supplementary file 1 [file mmc1.pdf]

EVIDENCE UPDATE FOR THE TREATMENT OF ANAPHYALXIS: Evidence to Decision (EtD) tables

Amy Dodd,[1]* Anna Hughes,[1]* Nicholas Sargant,[2] Andrew F Whyte,[3] Jasmeet Soar,[4#] Paul J. Turner[5#]

[1]Severn Deanery, UK; [2]Bristol Royal Hospital for Children, Bristol, UK; [3]University Hospitals Plymouth NHS Trust, Plymouth, UK; [4]North Bristol NHS Trust, Bristol, UK; [5]National Heart & Lung Institute, Imperial College London, UK

Table S1: Criteria that influence the strength and direction in the Evidence to Decision frameworks[6]

Criteria	How the factor influences the direction and strength of a recommendation
Problem	The problem is determined by the importance and frequency of the health care issue that is addressed (burden of disease, prevalence, or baseline risk). If the problem is of great importance, a strong recommendation is more likely.
Values and preferences	This describes how important health outcomes are to those affected, how variable they are and if there is uncertainty about this. Values and preferences or the importance of outcomes
Certainty in the evidence	The higher the certainty in the evidence, the more likely is a strong recommendation.
Health benefits and harms and burden and their balance	This requires an evaluation of the absolute effects of both the benefits and harms and their importance. The greater the net benefit or net harm, the more likely is a strong recommendation for or against the option.
Resource implications	This describes how resource intense an option is, if it is cost-effective and if there is incremental benefit. The more advantageous or clearly disadvantageous these resource implications are the more likely is a strong recommendation.
Equity	The greater the likelihood to reduce inequities or increase equity and the more accessible an option is, the more likely is a strong recommendation.
Acceptability	The greater the acceptability of an option to all or most stakeholders, the more likely is a strong recommendation.
Feasibility	The more feasible an option is, the more likely is a strong recommendation.

1. Is adrenaline effective in the treatment of anaphylaxis?

**POPULATION:**	Infants, children, adults and over 65s with suspected anaphylaxis.	**SETTING:**	Hospital and out-of-hospital setting
**INTERVENTION:**	Adrenaline by any route	**COMPARISON:**	No adrenaline
**MAIN OUTCOMES:**	1. Symptom resolution<br>2. Survival with good functional outcome, survival with complications		3. Occurrence of biphasic reaction<br>4. Biphasic reaction which prompts return visit to Emergency Department

ASSESSMENT

Desirable Effects — How substantial are the desirable anticipated effects?

JUDGEMENT	RESEARCH EVIDENCE	ADDITIONAL CONSIDERATIONS
Large	The **WAO 2015 Anaphylaxis Guideline update** states "International guidelines concur that epinephrine (adrenaline) is the medication of first choice in anaphylaxis because it is the only medication that reduces hospitalization and death." However, no actual evidence is cited for that statement. While national and international guidelines agree that the first-line treatment of anaphylaxis is intramuscular adrenaline, the underlying evidence base is limited, due to a lack of RCTs undertaken in patients having anaphylaxis (due to ethical concerns and widespread consensus that adrenaline is effective in treating allergic reactions and anaphylaxis).<br><br>• The original **WAO 2011 Anaphylaxis Guideline** notes that "the evidence base for prompt epinephrine injection in the initial treatment of anaphylaxis is stronger than the evidence base for the use of antihistamines and glucocorticoids in anaphylaxis. It consists of: observational studies performed in anaphylaxis, randomized-controlled clinical pharmacology studies in patients at risk for anaphylaxis but not experiencing it at the time of the investigation, studies in animal models of anaphylaxis, in vitro studies, and retrospective studies, including epidemiologic studies and fatality studies."<br>• **EAACI 2014 systematic review** reported 3 systematic reviews, none of which found any RCTs or quasi-RCTs, and concluded that there on the basis of "methodologically lower quality evidence from case-series and fatality registers, there is some evidence to support the use of adrenaline for the emergency management of anaphylaxis."<br>• The updated **EAACI 2020 systematic review** commented that "a number of reviews have examined the efficacy of adrenaline, but these mainly reported studies at high risk of bias. Our review only included comparative studies or consecutive case series with at least 20 participants and we identified no eligible studies comparing adrenaline versus no adrenaline in terms of mortality or most other outcomes".<br><br>There is little doubt that adrenaline can result in symptom resolution. However, in the largest series of fatal food anaphylaxis ever published, one third of fatalities received intramuscular adrenaline prior to arrest and yet still died (Pumphrey and Sturm, 2014). The situation is further confounded by a significant proportion (around 80%) of anaphylaxis reactions resolving without or despite no treatment with adrenaline (Noimark et al, 2012; Grabenhenrich et al, 2018) and a very low associated fatality rate (Nassiri et al, 2015) due to the fact the severe fatal outcomes in anaphylaxis are very rare (Umasunthar et al, 2013). Nonetheless, case series of refractory anaphylaxis reactions and animal models clearly show that *sufficient* adrenaline and fluid support is likely to be the issue, with severe reactions requiring more than just one or two doses of adrenaline for resolution. A case-control study of fatal versus non-fatal anaphylaxis would be helpful and achievable in generating the data needed to inform this area.<br><br>There is no evidence that adrenaline prevents biphasic reactions:<br><br>• The **EAACI 2020 systematic review** reported two case-control studies in children, but concluded "it is unclear whether adrenaline prevents biphasic anaphylactic reactions because the certainty of evidence is very low. One study found a non-statistically significant reduction of 9% and the other a significant reduction of 18% (odds ratio (OR) 0.08, 95% CI 0.014 to 0.43)."<br>• While the **2020 JTFPP** assessed whether the *timing* of adrenaline impacted on the occurrence of biphasic reactions, they did not evaluate the impact of *any* adrenaline.<br>• A systematic review and meta-analysis of 27 studies (2758 patients, 5% rate of biphasic reactions) reported no benefit of adrenaline treatment (pooled OR 0.91, 95% CI: 0.6 to 1.4) (Lee et al, 2015). This is consistent with data from the European Anaphylaxis Registry (7328 patients, 5% rate of biphasic reactions; OR 0.91, 95% CI 0.71-1.16) (Kraft et al, 2020).	**EAACI 2020** noted "adrenaline prophylaxis prior to snake bite anti-venom may reduce anaphylaxis. However, this evidence comes largely from Asia and may relate to types of anti-venoms that are not commonly used in other parts of the world"

Undesirable Effects
How substantial are the undesirable anticipated effects?

JUDGEMENT	RESEARCH EVIDENCE	ADDITIONAL CONSIDERATIONS
Varies	When used by the intramuscular route, there is minimal risk of adverse effects. Adrenaline given off protocol is associated with a high risk of adverse effects including death. Key areas for concern are the risk of overdose and cardiovascular complications.<br><br>The **EAACI Anaphylaxis Guideline (2014)** quotes the **WAO 2011 Guideline** and states: "There are no absolute contra-indications to treatment with adrenaline in a patient experiencing anaphylaxis; benefits outweigh the risks in the elderly and patients with pre-existing cardiovascular disease"<br><br>The WAO 2011 guideline further comments:<br>• "Serious adverse effects such as ventricular arrhythmias, hypertensive crisis, and pulmonary oedema potentially occur after an overdose of epinephrine by any route of administration. Typically, they are reported after intravenous epinephrine dosing; for example, overly rapid intravenous infusion, bolus administration, and dosing error."<br>• "Acute coronary syndromes (angina, myocardial infarction, arrhythmias) can also occur in untreated anaphylaxis in patients with known coronary artery disease, in those in whom subclinical coronary artery disease is unmasked, and even in patients (including children) without coronary artery disease in whom the symptoms are due to transient vasospasm"	

Certainty of evidence
What is the overall certainty of the evidence of effects?

Moderate	There are no RCTs or quasi RCTs comparing adrenaline treatment with placebo or no intervention. Evidence is therefore based on experience and observational data. Adrenaline is effective for symptoms of anaphylaxis and moderate certainty evidence that adrenaline improves outcomes (when used appropriately and in sufficient doses). While confidence in the effect estimate is limited, data from a systematic review and meta-analysis (including 36,557 anaphylaxis events) indicate that only 2.2% (95% CI 1.1 to 4.1%) of anaphylaxis reactions fail to respond to 2 doses of adrenaline (Patel et al, 2021). It is very unlikely that true effect of adrenaline is substantially different from this estimate. In contrast to morbidity data, there is low certainty evidence that adrenaline reduces mortality, due to the rarity of fatal outcomes in anaphylaxis. Overall, the certainty of evidence was rated as moderate, since fatal outcomes are exceedingly rare.	

Values
Is there important uncertainty about or variability in how much people value the main outcomes?

No important uncertainty or variability	Survival with good functional outcome is the desired outcome in anaphylaxis.	

Balance of effects
Does the balance between desirable and undesirable effects favour the intervention or the comparison?

Favours the intervention	Adrenaline is a potentially life-saving treatment; the benefits of administration outweigh the potential associated risks.	

Resources required and cost effectiveness
How large are the resource requirements (costs)? Does the cost-effectiveness of the intervention favour the intervention or the comparison?

Favours the intervention	Adrenaline is already widely used in clinical practice and as such resources will already be in place in hospital settings. No formal analysis undertaken.<br>The decision to use adrenaline autoinjectors (AAIs) in clinical settings would have cost implications.	

Equity, Acceptability, Feasibility
What would be the impact on health equity? Is the intervention acceptable to key stakeholders? Is the intervention feasible to implement?

Equity: probably increased<br><br>Acceptability: yes<br><br>Feasibility: yes	Ensure all patients have equal access to best anaphylaxis care in accordance with the NHS constitution<br><br>Adrenaline is already widely used in clinical practice, and accepted as the first-line treatment for anaphylaxis in all settings.	

	JUDGEMENT						
**PROBLEM**	No	Probably no	Probably yes	**Yes**		Varies	Don't know
**DESIRABLE EFFECTS**	Trivial	Small	Moderate	**Large**		Varies	Don't know
**UNDESIRABLE EFFECTS**	Large	Moderate	Small	Trivial		**Varies**	Don't know
**CERTAINTY OF EVIDENCE**	Very low	Low	**Moderate**	High			No included studies
**VALUES**	Important uncertainty or variability	Possibly important uncertainty or variability	Probably no important uncertainty or variability	**No important uncertainty or variability**			
**BALANCE OF EFFECTS**	Favours the comparison	Probably favours the comparison	Favours neither the intervention or comparator	Probably favours the intervention	**Favours the intervention**	Varies	Don't know
**RESOURCES REQUIRED**	Large costs	Moderate costs	**Negligible costs and savings**	Moderate savings	Large savings	Varies	Don't know
**CERTAINTY OF EVIDENCE OF REQUIRED RESOURCES**	Very low	Low	Moderate	High			**No included studies**
**COST EFFECTIVENESS**	Favours the comparison	Probably favours the comparison	Favours neither the intervention or comparator	Probably favours the intervention	**Favours the intervention**	Varies	No included studies
**EQUITY**	Reduced	Probably reduced	Probably no impact	**Probably increased**	Increased	Varies	Don't know
**ACCEPTABILITY**	No	Probably no	Probably yes	**Yes**		Varies	Don't know
**FEASIBILITY**	No	Probably no	Probably yes	**Yes**		Varies	Don't know

CONCLUSIONS

Recommendation

We recommend adrenaline as the first line treatment for anaphylaxis (strong recommendation, moderate certainty evidence)
Evidence is based on expert opinion and observational data from human studies and animal models which favours the benefits of adrenaline (which may be potentially lifesaving) over the possible adverse effects. While confidence in the effect estimate is limited, data from a systematic review and meta-analysis (including 36,557 anaphylaxis events) indicate that only 2.2% (95% CI 1.1 to 4.1%) of anaphylaxis reactions fail to respond to 2 doses of adrenaline (Patel, 2021). It is very unlikely that the true effect of adrenaline is substantially different from this estimate, and thus under GRADE EtD framework definitions (Table 2, main manuscript), the certainty over the response of anaphylaxis to adrenaline treatment was graded as moderate. The strong recommendation for adrenaline is based on the working group putting a high value on the evidence suggesting adrenaline is the most appropriate treatment to reduce morbidity, mortality and prevent harm, its universal use in existing international guidelines for anaphylaxis, and support from the public and stakeholders during the consultation process.

Other considerations

As highlighted by a Cochrane review (Sheikh et al, 2008), "although there is a need for randomized, double-blind, placebo-controlled clinical trials of high methodological quality in order to define the true extent of benefits from the administration of adrenaline in anaphylaxis, such trials are unlikely to be performed in individuals with anaphylaxis. Indeed, they might be unethical because prompt treatment with adrenaline is deemed to be critically important for survival in anaphylaxis. Also, such studies would be difficult to conduct because anaphylactic episodes usually occur without warning, often in a non-medical setting, and differ in severity both among individuals and from one episode to another in the same individual. Consequently, obtaining baseline measurements and frequent timed measurements might be difficult, or impossible, to obtain." A national registry of all anaphylaxis cases would be helpful to generate more evidence for the use of adrenaline to treat anaphylaxis, as RCTs are unlikely to be carried out.

2. What is the optimal timing of adrenaline in the treatment of anaphylaxis?

**POPULATION:**	Infants, children, adults and over 65 years with suspected anaphylaxis.	**SETTING:**	Hospital and out-of-hospital setting
**INTERVENTION:**	Administration of adrenaline by any route within 30mins of symptom onset	**COMPARISON:**	Delayed administration
**MAIN OUTCOMES:**	1. Symptom resolution<br>2. Survival with good functional outcome, survival with complications	3. Occurrence of biphasic reaction<br>4. Biphasic reaction which prompts return visit to Emergency Department	

ASSESSMENT

Desirable Effects — How substantial are the desirable anticipated effects?

JUDGEMENT	RESEARCH EVIDENCE	ADDITIONAL CONSIDERATIONS
Large	Evidence from case series including anaphylaxis fatalities suggests that early administration of adrenaline in out-of-hospital anaphylaxis is associated with improved outcomes.<br><br>The updated **EAACI 2020 systematic review** included 2 studies:<br>i. A retrospective review of 384 emergency department visits for food-induced anaphylaxis in children reported that "after adjusting for age, sex, and race, patients who received pre-hospital epinephrine remained at significantly decreased risk of hospitalization compared with those who received late epinephrine treatment [in the Emergency Department] (odds ratio 0.25 [95% CI, 0.12-0.49])." There was no impact on admissions to ICU. (Fleming et al, 2015).<br>ii. A case series by Liu et al (2020) "found that people who received adrenaline in hospital were no more likely to have a biphasic reaction than those who received the first dose of adrenaline before hospital once time to adrenaline administration was controlled for. Those who received adrenaline with a delay of more than 30 minutes from anaphylaxis onset were more likely to have a biphasic reaction (83% vs 60%, OR 3.39, 95% CI, 1.13 to 10.18, p = 0.02)."<br><br>The **2020 American Joint Task Force on Practice Parameters (JTFPP)** identified 8 retrospective case series that assessed the impact of timing of adrenaline on the occurrence of biphasic reactions. Unfortunately, the authors were unable to undertake a meta-analysis "since the authors provided interquartile range and median values and therefore this outcome could not be pooled together. Three of the eight studies showed delayed administration of epinephrine resulted in higher rates of biphasic anaphylaxis while the other five studies showed no statistical difference." One study by Lee et al. (2017) which included 872 anaphylaxis-related visits to an emergency department from 2008-2015 reported an OR of 2.29 (95%CI 1.09, 4.79) for biphasic reaction when the first dose of adrenaline was administrated more than 60 minutes after initial symptoms. The JTFPP concludes that "there does appear to be a trend to lower rates of biphasic reactions with earlier epinephrine administration following development of anaphylaxis…Prompt and adequate treatment of anaphylaxis appears central to reducing biphasic anaphylaxis risk. While early epinephrine in the setting of anaphylaxis is important, evidence suggests pre-emptive epinephrine before symptom onset is generally not a cost-effective strategy."	While there is widespread consensus that adrenaline should be used early to treat anaphylaxis, there is no evidence to suggest that pre-emptive use of adrenaline to treat mild symptoms to prevent progression to anaphylaxis is effective. There is at least one case report of a patient who was repeatedly administered adrenaline during an initially mild allergic reaction to brazil nut, which did not prevent progression to fatal respiratory arrest (Turner et al, 2016).

Undesirable Effects — How substantial are the undesirable anticipated effects?

JUDGEMENT	RESEARCH EVIDENCE	ADDITIONAL CONSIDERATIONS
Small	Treatment of more mild, non-anaphylaxis reactions is unlikely to be a concern in the hospital setting.	Concerns have been documented over the use (and efficacy) of adrenaline to *routinely* treat non-anaphylaxis reactions (Turner et al, 2016).

Certainty of evidence — What is the overall certainty of the evidence of effects?

JUDGEMENT	RESEARCH EVIDENCE	ADDITIONAL CONSIDERATIONS
Very low	The updated **EAACI 2020 systematic review** commented that "the most effective timing of adrenaline administration is unknown because the certainty of evidence is very low."	

Values — Is there important uncertainty about or variability in how much people value the main outcomes?

JUDGEMENT	RESEARCH EVIDENCE	ADDITIONAL CONSIDERATIONS
No important uncertainty or variability	Survival with good functional outcome is the desired outcome in anaphylaxis.	

Balance of effects — Does the balance between desirable and undesirable effects favour the intervention or the comparison?

JUDGEMENT	RESEARCH EVIDENCE	ADDITIONAL CONSIDERATIONS
Probably favours the intervention	Adrenaline is a potentially life-saving treatment; the benefits of early administration outweigh the potential associated risks, although concerns have been raised over the use of adrenaline to treat reactions that are clearly more mild and do not meet the criteria for anaphylaxis.	

Resources required and cost effectiveness — How large are the resource requirements (costs)? Does the cost-effectiveness of the intervention favour the intervention or the comparison?

JUDGEMENT	RESEARCH EVIDENCE	ADDITIONAL CONSIDERATIONS
Probably favours the intervention	Adrenaline is already widely used in clinical practice and as such resources will already be in place in hospital settings. No formal analysis undertaken.	The decision to use adrenaline autoinjectors (AAIs) in clinical settings would have cost implications.

Equity, Acceptability, Feasibility — What would be the impact on health equity? Is the intervention acceptable to key stakeholders? Is the intervention feasible to implement?

JUDGEMENT	RESEARCH EVIDENCE	ADDITIONAL CONSIDERATIONS
Equity: probably increased<br><br>Acceptability: yes<br><br>Feasibility: yes	Ensure all patients have equal access to best anaphylaxis care in accordance with the NHS constitution<br><br>Adrenaline is already widely used in clinical practice, and accepted as the first-line treatment for anaphylaxis in all settings.	

	JUDGEMENT						
**PROBLEM**	No	Probably no	Probably yes	**Yes**		Varies	Don't know
**DESIRABLE EFFECTS**	Trivial	Small	Moderate	**Large**		Varies	Don't know
**UNDESIRABLE EFFECTS**	Large	Moderate	**Small**	Trivial		Varies	Don't know
**CERTAINTY OF EVIDENCE**	**Very low**	Low	Moderate	High			No included studies
**VALUES**	Important uncertainty or variability	Possibly important uncertainty or variability	Probably no important uncertainty or variability	**No important uncertainty or variability**			
**BALANCE OF EFFECTS**	Favours the comparison	Probably favours the comparison	Favours neither the intervention or comparator	**Probably favours the intervention**	Favours the intervention	Varies	Don't know
**RESOURCES REQUIRED**	Large costs	Moderate costs	**Negligible costs and savings**	Moderate savings	Large savings	Varies	Don't know
**CERTAINTY OF EVIDENCE OF REQUIRED RESOURCES**	Very low	Low	Moderate	High			**No included studies**
**COST EFFECTIVENESS**	Favours the comparison	Probably favours the comparison	Favours neither the intervention or comparator	**Probably favours the intervention**	Favours the intervention	Varies	No included studies
**EQUITY**	Reduced	Probably reduced	Probably no impact	**Probably increased**	Increased	Varies	Don't know
**ACCEPTABILITY**	No	Probably no	Probably yes	**Yes**		Varies	Don't know
**FEASIBILITY**	No	Probably no	Probably yes	**Yes**		Varies	Don't know

CONCLUSIONS

Recommendation

Adrenaline should be administered early once symptoms of anaphylaxis have been recognized or suspected (weak recommendation, very low certainty evidence).

Adrenaline is a life-saving treatment for anaphylaxis and should be administered as soon as possible by non-clinical caregivers or health professional trained to recognize anaphylaxis and administer adrenaline via an appropriate device (AAI, pre-filled syringe, non pre-filled syringe).

Other considerations

Additional work is required to better assess the impact of early vs delayed administration of adrenaline on clinical outcomes.

3. What is the optimal route of adrenaline to treat anaphylaxis?

**POPULATION:**	Infants, children, adults and over 65s with suspected anaphylaxis.	**SETTING:**	Hospital and out-of-hospital setting
**INTERVENTION:**	Adrenaline by the intramuscular (IM) route	**COMPARISON:**	Adrenaline by an alternative route
**MAIN OUTCOMES:**	1. Symptom resolution<br>2. Pharmacokinetic profile (plasma concentration of adrenaline)	3. Safety / adverse events<br>4. Speed of administration	

ASSESSMENT

**Desirable Effects** How substantial are the desirable anticipated effects?		
**JUDGEMENT**	**RESEARCH EVIDENCE**	**ADDITIONAL CONSIDERATIONS**
Large	All international guidelines recommend intramuscular (IM) adrenaline for the initial treatment of anaphylaxis. However, as confirmed by an **EAACI 2020 systematic review**, "it is unclear whether different adrenaline administration routes affect outcomes because the certainty of evidence is very low." There are no trials comparing different routes of administration in patients undergoing acute reactions.<br>Comparing the IM to IV route, the **EAACI 2020** review identified a case series (children and adults) which found that "IV bolus administration was associated with a 13% increase in the incidence of adrenaline overdose and an 8% increase in the incidence of cardiovascular events compared with IM administration (Campbell et al. 2015) (very low certainty evidence).<br>Data from animal models demonstrate the superiority of the IV route in terms of pharmacokinetics, but found that bolus administration had little benefit once anaphylactic shock is established (Bautista et al, 2002). In a follow-on study, the authors demonstrated the superiority of IV infusion compared to bolus via the IM, IV or subcutaneous routes in causing haemodynamic improvement (Mink et al, 2014). Low dose IV adrenaline infusions been shown to be of benefit in case series of human anaphylaxis (Brown et al, 2004) and are included as the treatment of choice for refractory anaphylaxis in national guidelines in Australia and Spain (following initial IM adrenaline).<br>Comparing the IM to the subcutaneous route, the **EAACI 2020** review concluded that clinical trials in patients being given adrenaline outside the context of an allergic reaction show that "IM adrenaline was associated with an absolute increase of mean plasma adrenaline concentration (very low certainty); however, these studies may be confounded by using different injection sites (thigh versus arm), in addition to different depth of injection." A recent review has summarised more recent evidence for the intramuscular route over the subcutaneous route (Dreborg & Kim, 2021).<br>With respect to adrenaline given by the inhalational route, the **EAACI 2020** review identified two randomized trials and two nonrandomized trials (three in adults and one in children). Overall, "most studies found that inhalation did not deliver a therapeutically appropriate dose of adrenaline or reduce adverse effects compared to IM or subcutaneous injection or placebo (very low certainty)." **EAACI 2014 guideline** notes that "one caveat is stridor from laryngeal oedema where nebulized adrenaline (2–5 ml, 1mg/ml) can be used in addition to intramuscular adrenaline."<br>In terms of the practicalities of IM injection, the **EAACI 2020** review reported one trial in which untrained caregivers administered adrenaline without error more frequently using a prefilled syringe compared to an auto-injector (OR 4.07, 95%CI 1.29, 12.86; low certainty) (Suwan 2018). A similar study with radiologists found using an auto-injector reduced the time to administration by an average of 70 seconds compared to a syringe/ampoule, and resulted in fewer administration errors (very low certainty evidence) (Asch 2017). The main delay seems to be caused by the time taken (and potential for dosing errors) when drawing up adrenaline into a syringe rather than using a pre-filled syringe or auto-injector device (Simons et al, 2001).	In line with international resuscitation protocols, the intraosseous route is an alternative to the IV route in patients with anaphylactic shock.
**Undesirable Effects** How substantial are the undesirable anticipated effects?		
Varies	When using the IM adrenaline correctly, there is minimal risk of adverse effects. **EAACI 2014 guideline** states "the safety profile of IM adrenaline is excellent although patients may experience transient pallor, palpitations and headache" and cautions that while "patients who require repeated IM doses of adrenaline may benefit from an adrenaline infusion,… intravenous adrenaline in patients with adequate circulation may cause life-threatening hypertension, myocardial ischemia, and arrhythmias."	

	Fatalities have been reported due to IV adrenaline overdose used in the context of both anaphylaxis and non-anaphylaxis reactions. IV bolus administration is associated with higher rates of adrenaline overdose and cardiovascular adverse events compared to IM administration (Campbell et al. 2015). **WAO 2011 guideline** notes that “complications can occur regardless of route but are more common after IV administration or over rapid infusions”. A retrospective case review of 492 cases of anaphylaxis reported that “older patients with anaphylaxis were less likely to receive adrenaline injection. IM adrenaline appears safe in this population; however, the use of intravenous adrenaline should be avoided in older patients due to the potential of developing serious cardiac complications.” (Kawano et al, 2017). Takotsubo syndrome (cardiomyopathy) can occur following both IM and IV adrenaline dosing, but is more common with the IV route (Madias, 2016).	

Certainty of evidence
What is the overall certainty of the evidence of effects?

JUDGEMENT	RESEARCH EVIDENCE	ADDITIONAL CONSIDERATIONS
Low/variable	Low certainty over the route of administration. Data from a systematic review and meta-analysis (including 36,557 anaphylaxis events) indicate that only 2.2% (95% CI 1.1 to 4.1%) of anaphylaxis reactions fail to respond to 2 doses of adrenaline, the majority of which were delivered by the intramuscular route (Patel, 2021). Our confidence in the effect estimate is limited, but the true effect is unlikely be substantially different from the estimate of the effect. It is unclear whether different adrenaline administration routes affect outcomes because the certainty of evidence is very low. There are no trials comparing different routes of administration in patients undergoing acute reactions. However, use of adrenaline in other contexts indicates that the IM route is preferable to the IV route, so long as effectiveness is not compromised.	

Values
Is there important uncertainty about or variability in how much people value the main outcomes?

JUDGEMENT	RESEARCH EVIDENCE	ADDITIONAL CONSIDERATIONS
No important uncertainty or variability	Survival with good functional outcome is the desired outcome in anaphylaxis.	

Balance of effects
Does the balance between desirable and undesirable effects favour the intervention or the comparison?

JUDGEMENT	RESEARCH EVIDENCE	ADDITIONAL CONSIDERATIONS
Probably favours the intervention	IM adrenaline is favoured over other routes of administration for initial treatment of anaphylaxis, due to a favourable adverse event profile (including in those with cardiovascular co-morbidities) and concerns over the IV route in terms of potential for overdose and increased adverse events, particularly when used by those without specialist expertise.	

Resources required and cost effectiveness
How large are the resource requirements (costs)? Does the cost-effectiveness of the intervention favour the intervention or the comparison?

JUDGEMENT	RESEARCH EVIDENCE	ADDITIONAL CONSIDERATIONS
Probably favours the intervention	Adrenaline is already widely used in clinical practice and as such resources will already be in place in hospital settings. No formal analysis undertaken.	The decision to use adrenaline autoinjectors (AAIs) in clinical settings would have cost implications.

Equity, Acceptability, Feasibility
What would be the impact on health equity? Is the intervention acceptable to key stakeholders? Is the intervention feasible to implement?

Equity: probably increased<br><br>Acceptability: yes<br><br>Feasibility: yes	Ensure all patients have equal access to best anaphylaxis care in accordance with the NHS constitution<br><br>Adrenaline is already widely used in clinical practice, and accepted as the first-line treatment for anaphylaxis in all settings.	

	JUDGEMENT						
**PROBLEM**	No	Probably no	Probably yes	**Yes**		Varies	Don't know
**DESIRABLE EFFECTS**	Trivial	Small	Moderate	**Large**		Varies	Don't know
**UNDESIRABLE EFFECTS**	Large	Moderate	Small	Trivial		**Varies**	Don't know
**CERTAINTY OF EVIDENCE**	Very low	**Low**	Moderate	High			No included studies
**VALUES**	Important uncertainty or variability	Possibly important uncertainty or variability	Probably no important uncertainty or variability	**No important uncertainty or variability**			
**BALANCE OF EFFECTS**	Favours the comparison	Probably favours the comparison	Favours neither the intervention or comparator	**Probably favours the intervention**	Favours the intervention	Varies	Don't know
**RESOURCES REQUIRED**	Large costs	Moderate costs	**Negligible costs and savings**	Moderate savings	Large savings	Varies	Don't know
**CERTAINTY OF EVIDENCE OF REQUIRED RESOURCES**	Very low	Low	Moderate	High			**No included studies**
**COST EFFECTIVENESS**	Favours the comparison	Probably favours the comparison	Favours neither the intervention or comparator	**Probably favours the intervention**	Favours the intervention	Varies	No included studies
**EQUITY**	Reduced	Probably reduced	Probably no impact	**Probably increased**	Increased	Varies	Don't know
**ACCEPTABILITY**	No	Probably no	Probably yes	**Yes**		Varies	Don't know
**FEASIBILITY**	No	Probably no	Probably yes	**Yes**		Varies	Don't know

CONCLUSIONS

Recommendation

1. **The intramuscular (IM) route is recommended for initial adrenaline treatment for anaphylaxis (strong recommendation, low certainty evidence).**
2. **The intravenous (IV) route is not recommended for initial management of anaphylaxis, except in the perioperative setting (as an alternative to IM adrenaline) by those skilled and experienced in its use (good practice statement).**
   - **In such circumstances, adrenaline should preferably be administered as an IV infusion and not as a bolus dose (weak recommendation, very low certainty evidence).**
3. **Titrate the administration of adrenaline (by any route) against clinical response (strong recommendation, very low certainty evidence).**

Although the level of certainty of evidence was low, the working group agreed with other guidelines (such as EAACI 2021) that “given the totality of the evidence and clinical experience over many decades, … a strong recommendation for the use of IM adrenaline was appropriate” (Muraro, 2021). The working group justified its strong recommendation for the IM route, as it placed a high value on the relative ease and safety of IM adrenaline by a wide variety of healthcare staff, and the wide acceptance of the IM route, both in clinical and non-clinical settings (including by patients for self-administration using an autoinjector device). Titration of the dose of adrenaline to clinical response is standard practice to minimise the risks of excessive adrenaline, and forms the basis of the strong recommendation for this.

Intravenous (IV) adrenaline must only be used in certain specialist settings and only by those skilled and experienced in its use. It is not recommended for initial management of anaphylaxis, except in the context of peri-operative anaphylactic shock where both the IM and IV routes can be used by those with appropriate experience.

IV adrenaline infusions form the basis of treatment for refractory anaphylaxis: seek expert help early in patients with refractory anaphylaxis.

Other considerations

- In line with international resuscitation protocols, the interosseous route is an alternative to the IV route in patients with anaphylactic shock if intravenous access cannot be rapidly obtained.

4. What is the optimal dose for intramuscular adrenaline in the treatment of anaphylaxis?

**POPULATION:**	Infants, children, adults and over 65s with suspected anaphylaxis.	**SETTING:**	Hospital and out-of-hospital setting
**INTERVENTION:**	Intramuscular adrenaline	**COMPARISON:**	N/A
**MAIN OUTCOMES:**	1. Resolution of symptoms<br>2. Survival with good functional outcome, survival with complications		

ASSESSMENT

Desirable Effects
How substantial are the desirable anticipated effects?

JUDGEMENT	RESEARCH EVIDENCE	ADDITIONAL CONSIDERATIONS
Large	The **WAO 2011 anaphylaxis guideline** recommends IM adrenaline at a dose of 0.01 mg/kg as "effective and safe in the initial treatment of anaphylaxis." The guideline further suggests that in scenarios such as anaphylactic shock or peri-arrest, this low dose is unlikely to be effective. In such circumstances, adrenaline "needs to be given by slow intravenous infusion, ideally with the dose titrated according to non-invasive continuous monitoring of cardiac rate and function. If cardiac arrest is imminent or has already occurred, an intravenous bolus dose of adrenaline is indicated." Many guidelines (including WAO 2020 guidance and EAACI 2014 and RCUK) simplify the dosing regimen to age categories which match the licensed doses used for auto-injectors. This pragmatic approach seems to be effective and safe, compared to a dosing regime based on body mass.<br><br>Coronial inquests have identified that the use of certain brands of adrenaline auto-injectors for anaphylaxis result in a substantial under-dose compared to doses recommended for use by healthcare professionals, which could be a contributory factor to fatal outcomes. For example, administering a dose of 300 microgram by auto-injector in an individual ≥50kg results in a 40% under-dosing. Anecdotal data provides some further evidence, in that some individuals who have required 2 adrenaline doses of 300 mcg by auto-injector respond to 500mcg (given by auto-injector) at subsequent anaphylaxis reactions (although there are clearly significant potential confounders in comparing the treatment response at different reaction events).<br><br>There are no trials to identify the optimal dose of adrenaline for use in anaphylaxis. Dosing recommendations have been extrapolated from those used for other presentations where adrenaline is indicated. A randomized double-blind parallel-group study in 10 children (weight 15-30 kg) assessed the pharmaco-kinetics of 150 vs 300 mcg IM adrenaline. Unfortunately, children allocated to 300 mcg had a significantly greater body mass than those receiving 150mcg (mean 25.4kg (range 21.5-30) versus 18.0kg (range 16-20.4), although dosing per kg between the two groups was not statistically different. Those receiving 300 mcg had a more favourable absorption profile and effect on blood pressure, but also a higher incidence of (mild) adverse events (Simons et al, 2002).<br><br>In contrast, a single-blinded cross-over RCT in 12 teenagers at risk of food-anaphylaxis suggested that a 500mcg dose of adrenaline (given by auto-injector) had a more favourable pharmacokinetic and pharmacodynamic profile compared to 300mcg dose given using an auto-injector of the same brand (and thus identical needle length and mechanism). Importantly, this study also demonstrated that using plasma adrenaline as an outcome in these studies may not be an appropriate measure, and that haemodynamic outcomes (including stroke volume and cardiac output) are more relevant (Patel et al, 2019). Data from two commercially-sponsored cross-over RCTs are described in the approved Summary of Product Characteristics (SmPCs) for Emerade and Anapen; the latter has been published in the peer-reviewed literature (Duvauchelle 2018). These also indicate that a 500mcg dose given by needle/syringe results in higher plasma levels of adrenaline that 300mcg; how this impacts on clinical response has not been tested.<br><br>Finally, in an observational study of peanut-allergic adults undergoing anaphylaxis in hospital at supervised food challenge, Turner et al (2020) reported that "IM injection with adrenaline had limited impact in reversing the decrease in stroke volume caused by peanut-induced anaphylaxis. These data question the effectiveness of [a single dose of] intramuscular epinephrine alone to treat cardiovascular compromise during anaphylaxis."	

JUDGEMENT	RESEARCH EVIDENCE	ADDITIONAL CONSIDERATIONS
**Undesirable Effects** How substantial are the undesirable anticipated effects?		
Small	While overdose of adrenaline via the IV route is associated with a higher rate of adverse events, this may not hold true for IM adrenaline. In a single-blinded cross-over RCT in 12 teenagers at risk of food-anaphylaxis, a 500mcg dose of adrenaline (given by auto-injector) was associated with fewer mild adverse events than 300mcg dose given using the same auto-injector device. There were no serious adverse events with either dose (Patel et al, 2019).	
**Certainty of evidence** What is the overall certainty of the evidence of effects?		
Low	There is little evidence as to the optimal dose of IM adrenaline for anaphylaxis. In particular, studies often use plasma adrenaline levels as an outcome measure but there is no data to support the optimal plasma adrenaline level for anaphylaxis management. Furthermore, a recent study has cast significant doubt as to whether absorption profile for IM adrenaline is an appropriate surrogate outcome measure (Patel 2019).<br><br>Nonetheless, the current dosing regime is recommended in all international guidelines and there is significant (but low certainty) data that the current recommended doses of 0.01 mg/kg (max 500mcg) given every 5-15 minutes and repeated as needed, according to response, is appropriate and effective for resolution of symptoms.<br><br>Four small cross-over RCTs have been published which compare different doses of adrenaline: one in children of weight 15-30kg, comparing 150 to 300 microgram IM injection (Simons et al, 2002); and three comparing 300 to 500 microgram given IM by needle/syringe in teenagers or adults (Duvauchelle 2018; Emerade SmPC; Patel 2019). In all 4 studies, the larger dose had a more favourable absorption profile, however how this impacts on clinical response in patients actually experiencing anaphylaxis has not been assessed (the studies were all conducted outside the context of an allergic reaction).	
**Values** Is there important uncertainty about or variability in how much people value the main outcomes?		
No important uncertainty or variability	Survival with good functional outcome is the desired outcome in anaphylaxis.	
**Balance of effects** Does the balance between desirable and undesirable effects favour the intervention or the comparison?		
Probably favours the intervention	Current dosing recommendations for IM adrenaline are effective and safe, so long as the need for further adrenaline is titrated to clinical response and escalation to intravenous adrenaline infusion with appropriate expertise is implemented for refractory reactions.	
**Resources required and cost effectiveness** How large are the resource requirements (costs)? Does the cost-effectiveness of the intervention favour the intervention or the comparison?		
Probably favours the intervention	Adrenaline is already widely used in clinical practice and as such resources will already be in place in hospital settings. No formal analysis undertaken.	The decision to use adrenaline autoinjectors (AAIs) in clinical settings would have cost implications.
**Equity, Acceptability, Feasibility** What would be the impact on health equity? Is the intervention acceptable to key stakeholders? Is the intervention feasible to implement?		
Equity: probably increased<br><br>Acceptability: yes<br><br>Feasibility: yes	Ensure all patients have equal access to best anaphylaxis care in accordance with the NHS constitution<br><br>Adrenaline is already widely used in clinical practice, and accepted as the first-line treatment for anaphylaxis in all settings.	

	JUDGEMENT						
**PROBLEM**	No	Probably no	Probably yes	**Yes**		Varies	Don't know
**DESIRABLE EFFECTS**	Trivial	Small	Moderate	**Large**		Varies	Don't know
**UNDESIRABLE EFFECTS**	Large	Moderate	**Small**	Trivial		Varies	Don't know
**CERTAINTY OF EVIDENCE**	Very low	**Low**	Moderate	High			No included studies
**VALUES**	Important uncertainty or variability	Possibly important uncertainty or variability	Probably no important uncertainty or variability	**No important uncertainty or variability**			
**BALANCE OF EFFECTS**	Favours the comparison	Probably favours the comparison	Favours neither the intervention or comparator	**Probably favours the intervention**	Favours the intervention	Varies	Don't know
**RESOURCES REQUIRED**	Large costs	Moderate costs	**Negligible costs and savings**	Moderate savings	Large savings	Varies	Don't know
**CERTAINTY OF EVIDENCE OF REQUIRED RESOURCES**	Very low	Low	Moderate	High			**No included studies**
**COST EFFECTIVENESS**	Favours the comparison	Probably favours the comparison	Favours neither the intervention or comparator	**Probably favours the intervention**	Favours the intervention	Varies	No included studies
**EQUITY**	Reduced	Probably reduced	Probably no impact	**Probably increased**	Increased	Varies	Don't know
**ACCEPTABILITY**	No	Probably no	Probably yes	**Yes**		Varies	Don't know
**FEASIBILITY**	No	Probably no	Probably yes	**Yes**		Varies	Don't know

CONCLUSIONS

Recommendation

Intramuscular adrenaline should be administered at the following doses: (strong recommendation, low certainty evidence) on the basis of these doses being established in international guidelines for over 20 years:

ADULTS: 0.5 mg (500 micrograms) IM = 0.5 mL of 1mg/ml (1:1000) adrenaline
CHILDREN: >12 years: 0.5 mg (500 micrograms) IM = 0.5 mL of 1mg/ml (1:1000) adrenaline i.e. same as adult dose (300 micrograms (0.3 mL) if child is small or prepubertal)
6 – 12 years: 0.3mg (300 micrograms) IM = 0.3 mL of 1mg/ml (1:1000) adrenaline
6 months – 6 years: 0.15mg (150 micrograms) IM = 0.15 mL of 1mg/ml (1:1000) adrenaline
< 6 months: 100-150 micrograms IM = 0.1- 0.15 mL of 1mg/ml (1:1000) adrenaline

The working group concluded that while the certainty of evidence with respect to dose is low, these doses have been widely used internationally for many decades, and thus a strong recommendation was appropriate. In addition, we have not identified any new evidence to change this widely accepted approach.

Other considerations

- Administration of adrenaline (by any route) needs to be titrated against clinical response.
- In the peri-operative setting, it may be appropriate for IV bolus adrenaline to be given (as an alternative to IM adrenaline) depending on the available experience of the anaesthetic team.
- Where there is a suboptimal response to 2 doses of adrenaline (by IM or IV route), seek senior support as to establishing an adrenaline infusion to provide more appropriate inotropic and vasopressor support in the context of refractory anaphylaxis.

5. Is adrenaline effective in the treatment of anaphylaxis reactions refractory to initial treatment with adrenaline?

**POPULATION:**	Infants, children, adults and over 65s with suspected anaphylaxis.	**SETTING:**	Hospital and out-of-hospital setting
**INTERVENTION:**	Adrenaline by any route	**COMPARISON:**	Other interventions
**MAIN OUTCOMES:**	1. Resolution of symptoms<br>2. Survival with good functional outcome, survival with complications		

ASSESSMENT

Desirable Effects — How substantial are the desirable anticipated effects?

JUDGEMENT	RESEARCH EVIDENCE	ADDITIONAL CONSIDERATIONS
Large	International guidelines agree that IM adrenaline should be repeated where symptoms of anaphylaxis persist. Most suggest further IM doses of adrenaline every 5-15 minutes, although the rationale for waiting longer than 5 minutes is unclear given that the plasma half-life of adrenaline is 2-3 minutes (although this is likely to be prolonged through intramuscular injection).<br><br>An observational study in peanut-allergic adults undergoing anaphylaxis at supervised food challenge reported that "IM injection with adrenaline had limited impact in reversing the decrease in stroke volume caused by peanut-induced anaphylaxis. These data question the effectiveness of [a single dose of] IM adrenaline alone to treat cardiovascular compromise during anaphylaxis." (Turner et al, 2020).<br><br>Case series of refractory anaphylaxis reactions and data from animal models indicate that a poor response to adrenaline is likely due to *insufficient* adrenaline delivery, with severe reactions requiring more than just one or two doses of IM adrenaline for resolution. Data from animal models demonstrate the superiority of the IV route for adrenaline in terms of pharmacokinetics, but found that bolus administration had little benefit once anaphylactic shock is established (Bautista et al, 2002). In a follow-on study, the authors demonstrated the superiority of IV infusion compared to bolus via the IM, IV or subcutaneous routes in causing haemodynamic improvement (Mink et al, 2004). Low dose IV adrenaline infusions been shown to be of benefit in case series of human anaphylaxis (Brown et al, 2004) and are included as the treatment of choice for refractory anaphylaxis in national guidelines in Australia and Spain (following initial IM adrenaline).<br><br>**WAO 2011 guideline** notes that "Patients experiencing hypotension or shock refractory to basic initial treatment, including intravenous fluid resuscitation, require intravenous epinephrine and, sometimes, an additional intravenous vasopressor or other medication. No clear superiority of dopamine, dobutamine, norepinephrine, phenylephrine, or vasopressin (either added to epinephrine alone, or compared with one another), has been demonstrated in clinical trials.". The ASCIA 2020 Guideline recommends consideration of other vasopressors or inotropes only if an IV adrenaline infusion is ineffective. With the exception of glucagon, the **EAACI 2014 guideline** and **WAO 2020 guidance** do not mention other vasopressors or inotropes, but recommend the use of intravenous adrenaline infusion in patients whose reactions are refractory to treatment with IM adrenaline.<br><br>Research using an animal model of anaphylaxis have demonstrated that early treatment with adrenaline followed by continuous adrenaline or vasopressin infusion is superior to vasopressin alone (Dewachter et al, 2007; Zheng et al, 2015). The authors conclude that adrenaline must be considered the first-line drug to treat anaphylactic shock (Dewachter et al, 2007).	

Undesirable Effects — How substantial are the undesirable anticipated effects?

JUDGEMENT	RESEARCH EVIDENCE	ADDITIONAL CONSIDERATIONS
Varies	Fatalities have been reported due to IV adrenaline overdose used in the context of both anaphylaxis and non-anaphylaxis reactions. IV bolus administration is associated with higher rates of adrenaline overdose and cardiovascular adverse events compared to IM administration (Campbell et al. 2015). WAO 2011 guideline notes that "complications can occur regardless of route but are more common after IV administration or over rapid infusions". Such concerns need to be balanced against the risk of death in refractory reactions. Reassuringly, the use of low dose intravenous adrenaline infusions appears to both effective and safe in refractory reactions (Brown et al, 2004; Alviani et al, 2020).	

	A retrospective review of 492 anaphylaxis cases reported “older patients with anaphylaxis were less likely to receive adrenaline injection. IM adrenaline appears safe in this population; the use of IV adrenaline should be avoided in older patients due to the potential of developing serious cardiac complications.” (Kawano et al, 2017). Takotsubo syndrome (cardiomyopathy) can occur following both IM and IV adrenaline dosing, but is more common with the IV route (Madias, 2016).	
**Certainty of evidence** What is the overall certainty of the evidence of effects?		
**JUDGEMENT**	**RESEARCH EVIDENCE**	**ADDITIONAL CONSIDERATIONS**
Low	While there are no data from RCTs or quasi-RCTs, evidence of case series in humans and animal models indicate the efficacy of parenteral adrenaline in the treatment of refractory reactions when titrated carefully against clinical response (to minimise adverse effects of treatment). Further research is very likely to have an important impact on the confidence in the estimate of effect, and is likely to change the estimate.	
**Values** Is there important uncertainty about or variability in how much people value the main outcomes?		
**JUDGEMENT**	**RESEARCH EVIDENCE**	**ADDITIONAL CONSIDERATIONS**
No important uncertainty or variability	The main outcome is symptom resolution and survival with good functional outcome – this is universally highly valued.	
**Balance of effects** Does the balance between desirable and undesirable effects favour the intervention or the comparison?		
**JUDGEMENT**	**RESEARCH EVIDENCE**	**ADDITIONAL CONSIDERATIONS**
Probably favours the intervention	Use of adrenaline to treat refractory reactions (with dose titrated to clinical response) is potentially lifesaving, and these benefits outweigh the potential risks.	Expert support should be sought early in the management of anaphylaxis refractory to initial treatment with IM adrenaline.
**Resources required and cost effectiveness** How large are the resource requirements (costs)? Does the cost-effectiveness of the intervention favour the intervention or the comparison?		
**JUDGEMENT**	**RESEARCH EVIDENCE**	**ADDITIONAL CONSIDERATIONS**
Resources: Varies<br><br>Cost effectiveness: Probably favours the intervention	Adrenaline is already widely used in clinical practice and as such resources will already be in place in hospital settings. Multiple doses of adrenaline are associated with a prolonged period of observation and hospital admission, but this needs to be considered in the context of alternative outcomes including hypoxic brain injury and death due to severe anaphylaxis. Costs associated with admission and observation will vary according to setting. No formal analysis undertaken.	Early effective treatment is likely to reduce downstream costs.
**Equity, Acceptability, Feasibility** What would be the impact on health equity? Is the intervention acceptable to key stakeholders? Is the intervention feasible to implement?		
Equity: probably increased<br><br>Acceptability: yes<br><br>Feasibility: yes	Ensure all patients have equal access to best anaphylaxis care in accordance with the NHS constitution<br><br>Adrenaline is already widely used in clinical practice, and accepted as the first-line treatment for anaphylaxis in all settings. International guidelines increasingly promote the use of low dose intravenous infusions for reactions refractory to initial treatment. This is likely to improve access to these protocols in patients experiencing severe anaphylaxis.	

	JUDGEMENT						
**PROBLEM**	No	Probably no	Probably yes	**Yes**		Varies	Don't know
**DESIRABLE EFFECTS**	Trivial	Small	Moderate	**Large**		Varies	Don't know
**UNDESIRABLE EFFECTS**	Large	Moderate	Small	Trivial		**Varies**	Don't know
**CERTAINTY OF EVIDENCE**	Very low	**Low**	Moderate	High			No included studies
**VALUES**	Important uncertainty or variability	Possibly important uncertainty or variability	Probably no important uncertainty or variability	**No important uncertainty or variability**			
**BALANCE OF EFFECTS**	Favours the comparison	Probably favours the comparison	Favours neither the intervention or comparator	**Probably favours the intervention**	Favours the intervention	Varies	Don't know
**RESOURCES REQUIRED**	Large costs	Moderate costs	Negligible costs and savings	Moderate savings	Large savings	**Varies**	Don't know
**CERTAINTY OF EVIDENCE OF REQUIRED RESOURCES**	Very low	Low	Moderate	High			**No included studies**
**COST EFFECTIVENESS**	Favours the comparison	Probably favours the comparison	Favours neither the intervention or comparator	**Probably favours the intervention**	Favours the intervention	Varies	No included studies
**EQUITY**	Reduced	Probably reduced	Probably no impact	**Probably increased**	Increased	Varies	Don't know
**ACCEPTABILITY**	No	Probably no	Probably yes	**Yes**		Varies	Don't know
**FEASIBILITY**	No	Probably no	Probably yes	**Yes**		Varies	Don't know

CONCLUSIONS

Recommendation

1. **Subsequent doses of adrenaline should be given every 5 minutes, titrated to clinical response, in patients whose symptoms are refractory to initial treatment (weak recommendation, very low certainty evidence).**
2. **Where respiratory and/or cardiovascular features of anaphylaxis persist despite 2 appropriate doses of adrenaline (administered (by IM or IV route), seek urgent senior support (e.g. from experienced critical care clinicians) to establish an intravenous adrenaline infusion to treat refractory anaphylaxis (strong recommendation, low certainty evidence).**
3. **Low dose intravenous adrenaline infusions appear to be effective and safe to treat refractory anaphylaxis (weak recommendation, very low certainty evidence).**

Given the potential risks of using intravenous adrenaline infusion by those without the necessary expertise and support, and evidence supporting the use of intravenous adrenaline infusions to treat refractory reactions, the working group made a strong recommendation that urgent senior support is obtained to establish an intravenous adrenaline infusion to treat refractory anaphylaxis.

Other considerations

- To facilitate tissue delivery of adrenaline, patients experiencing refractory reactions should also receive an IV fluid bolus using a crystalloid.
- Intravenous adrenaline infusions are important in the management of all aspects of anaphylaxis and not only cardiovascular shock, and should thus be used first-line for refractory reactions. Other inotropes or vasopressors may be indicated in those with persistent systemic symptoms despite a lack of evidence, in accordance with the management of other shock states.

6. Are intravenous fluids effective as an adjuvant treatment for anaphylaxis?

**POPULATION:**	Infants, children, adults and over 65s with suspected anaphylaxis.	**SETTING:**	Hospital and out-of-hospital setting
**INTERVENTION:**	Intravenous fluids as an adjuvant for anaphylaxis	**COMPARISON:**	No intravenous fluids
**MAIN OUTCOMES:**	1. Resolution of symptoms<br>2. Survival with good functional outcome, survival with complications		

ASSESSMENT

Desirable Effects
How substantial are the desirable anticipated effects?

JUDGEMENT	RESEARCH EVIDENCE	ADDITIONAL CONSIDERATIONS
Large	Evidence from observational studies and animal models suggests that anaphylactic shock occurs as a consequence of a profound reduction in venous tone and fluid extravasation. Allergic mediators can also impair cardiac function. This results in a mix of hypovolemic, distributive and possibly cardiogenic shock, which combine to reduced venous return.<br><br>A recent study in peanut-allergic adults reported that a decrease in stroke volume occurs even during non-anaphylaxis reactions, but in most individuals cardiac output is maintained due to a compensatory tachycardia (Ruiz Garcia et al, 2020). A related study in the same cohort reported that "IM injection with adrenaline had limited impact in reversing the decrease in stroke volume caused by peanut-induced anaphylaxis. These data question the effectiveness of [a single dose of] intramuscular epinephrine alone to treat cardiovascular compromise during anaphylaxis and support the need for guidelines to incorporate effective adjuvant treatments in addition to intramuscular epinephrine in the management of refractory anaphylaxis." Administration of 500-1000ml of crystalloid had a greater effect on restoring venous return than a single dose of intramuscular adrenaline. Data from large case series and the European Anaphylaxis Registry indicate that a significant proportion (around 80%) of anaphylaxis reactions resolve without or despite no treatment with adrenaline (Noimark et al, 2012; Grabenhenrich et al, 2018). Together, these data suggest that poor outcomes in anaphylaxis may occur as a result of a failure of the host to compensate for the allergic reaction.<br><br>The **EAACI 2014 Anaphylaxis guideline** recommends, on the basis of expert consensus, that "Intravenous fluids should be administered to patients with cardiovascular instability, as adrenaline may not be effective without restoring the circulatory volume. This is consistent with the **WAO 2020 Anaphylaxis Guidance**.<br><br>However, given new data which suggests that reduced venous return can occur even in the absence of cardiovascular instability, it would seem prudent to administer an IV fluid bolus in all case of anaphylaxis refractory to initial treatment, irrespective of whether there is evidence of cardiovascular instability which may be a late sign of decompensation. The rationale is that the resulting restoration in circulating volume may aid adrenaline delivery and hasten resolution of symptoms.	

Undesirable Effects
How substantial are the undesirable anticipated effects?

JUDGEMENT	RESEARCH EVIDENCE	ADDITIONAL CONSIDERATIONS
Varies	Fluid overload is the main undesirable effect of IV fluids. A single bolus of IV crystalloid is unlikely to cause overload in the context of anaphylactic shock or refractory anaphylaxis.<br><br>**WAO 2011 Guideline** recommends "the rate of administration should be titrated according to the blood pressure, cardiac rate and function, and urine output. All patients receiving such treatment should be monitored for volume overload"	

**Certainty of evidence** What is the overall certainty of the evidence of effects?		
**JUDGEMENT**	**RESEARCH EVIDENCE**	**ADDITIONAL CONSIDERATIONS**
Very low	Previous recommendations are based on expert consensus, and as highlighted by the EAACI 2014 Guideline, there was "no evidence from primary studies for other potential treatments [for anaphylaxis], such as fluid replacement," although "intravenous fluids [crystalloids] should be administered to patients with cardiovascular instability, as adrenaline may not be effective without restoring the circulatory volume". A more recent observational study supports the use of intravenous fluids in refractory anaphylaxis. The evidence base for fluid therapy in shocked states is low (NICE Clinical Guidelines 174, 2017).	

**Values** Is there important uncertainty about or variability in how much people value the main outcomes?		
**JUDGEMENT**	**RESEARCH EVIDENCE**	**ADDITIONAL CONSIDERATIONS**
No important uncertainty or variability	Survival with good functional outcome is the desired outcome in anaphylaxis.	

**Balance of effects** Does the balance between desirable and undesirable effects favour the intervention or the comparison?		
**JUDGEMENT**	**RESEARCH EVIDENCE**	**ADDITIONAL CONSIDERATIONS**
Probably favours the intervention	Judicious use of IV fluids, titrated to clinical response, is potentially life-saving.	

**Resources required and cost effectiveness** How large are the resource requirements (costs)? Does the cost-effectiveness of the intervention favour the intervention or the comparison?		
**JUDGEMENT**	**RESEARCH EVIDENCE**	**ADDITIONAL CONSIDERATIONS**
Negligible costs and savings	Crystalloids are widely available and are of negligible cost. No formal analysis undertaken.	

**Equity, Acceptability, Feasibility** What would be the impact on health equity? Is the intervention acceptable to key stakeholders? Is the intervention feasible to implement?		
**JUDGEMENT**	**RESEARCH EVIDENCE**	**ADDITIONAL CONSIDERATIONS**
Equity:<br>probably increased<br><br>Acceptability: yes<br><br>Feasibility: yes	Ensure all patients have equal access to best anaphylaxis care in accordance with the NHS constitution<br><br>IV fluids are already widely used in clinical practice.	

	JUDGEMENT						
PROBLEM	No	Probably no	Probably yes	**Yes**		Varies	Don't know
DESIRABLE EFFECTS	Trivial	Small	Moderate	**Large**		Varies	Don't know
UNDESIRABLE EFFECTS	Large	Moderate	Small	Trivial		**Varies**	Don't know
CERTAINTY OF EVIDENCE	**Very low**	Low	Moderate	High			No included studies
VALUES	Important uncertainty or variability	Possibly important uncertainty or variability	Probably no important uncertainty or variability	**No important uncertainty or variability**			
BALANCE OF EFFECTS	Favours the comparison	Probably favours the comparison	Favours neither the intervention or comparator	**Probably favours the intervention**	Favours the intervention	Varies	Don't know
RESOURCES REQUIRED	Large costs	Moderate costs	**Negligible costs and savings**	Moderate savings	Large savings	Varies	Don't know
CERTAINTY OF EVIDENCE OF REQUIRED RESOURCES	Very low	Low	Moderate	High			**No included studies**
COST EFFECTIVENESS	Favours the comparison	Probably favours the comparison	Favours neither the intervention or comparator	**Probably favours the intervention**	Favours the intervention	Varies	No included studies
EQUITY	Reduced	Probably reduced	Probably no impact	**Probably increased**	Increased	Varies	Don't know
ACCEPTABILITY	No	Probably no	Probably yes	**Yes**		Varies	Don't know
FEASIBILITY	No	Probably no	Probably yes	**Yes**		Varies	Don't know

CONCLUSIONS

Recommendation

1. **In the presence of anaphylaxis with haemodynamic compromise, intravenous (IV) crystalloid fluids should be infused (weak recommendation, very low certainty evidence).**

2. **For anaphylaxis refractory to initial treatment with adrenaline, an IV fluid bolus (crystalloid) is recommended as an adjunct to improve drug distribution (weak recommendation, very low certainty evidence).**

Other considerations

The evidence base for fluid therapy in shocked states is low (NICE Clinical Guidelines 174, 2017).

7. Are antihistamines effective in the treatment of anaphylaxis?

**POPULATION:**	Infants, children, adults and over 65s with suspected anaphylaxis.	**SETTING:**	Hospital and out-of-hospital setting
**INTERVENTION:**	Antihistamine	**COMPARISON:**	No antihistamine
**MAIN OUTCOMES:**	1. Resolution of symptoms<br>2. Survival with good functional outcome, survival with complications		3. Occurrence of biphasic reaction<br>4. Biphasic reaction which prompts return visit to Emergency Department

ASSESSMENT

Desirable Effects — How substantial are the desirable anticipated effects?

JUDGEMENT	RESEARCH EVIDENCE	ADDITIONAL CONSIDERATIONS
Trivial effect in the resolution of anaphylaxis<br><br>Moderate effect in terms of action on skin symptoms post-anaphylaxis	The available evidence does not suggest there are substantial desirable anticipated effects (in terms of clinical improvement / survival) with the use of antihistamines in the treatment of anaphylaxis.<br>• **EAACI 2014 Anaphylaxis guideline** states that "Oral H1- (& H2)-antihistamines may relieve cutaneous symptoms of anaphylaxis, but are not of benefit in the treatment of anaphylaxis itself." They are a third-line intervention in the acute management of anaphylaxis.<br>• **2015 update of the evidence base: World Allergy Organization anaphylaxis guideline** notes that "H1-antihistamines [and] H2-antihistamines … are 2nd-line or even 3rd-line medications in anaphylaxis. These medications are not life saving and should not be used as initial or sole treatment"<br>• **ASCIA 2020 Guideline for acute management of anaphylaxis** states that "antihistamines have no role in treating or preventing respiratory or cardiovascular symptoms of anaphylaxis", and further cautions against the use of "oral sedating antihistamines as side effects (drowsiness or lethargy) may mimic some signs of anaphylaxis"<br>• **WAO 2020 Anaphylaxis Guidance** notes that "Antihistamines are now a third line treatment in some guidelines due to concern that their administration can delay more urgent measures such as repeated administration of parenteral epinephrine."<br>• The **2020 American Joint Task Force on Practice Parameters (JTFPP)** recommended against antihistamines as an intervention to prevent biphasic anaphylaxis based on very low-quality evidence (conditional recommendation).	**EAACI 2020:** No strong evidence to support use of antihistamines prior to snake anti-venom to prevent anaphylaxis.<br>**2020 JTFPP** notes that "based on very low-quality evidence, we suggest in favour of the administration of antihistamines as an intervention to prevent anaphylaxis in patients undergoing aeroallergen rush immunotherapy … or to prevent anaphylaxis or infusion related reactions when indicated for specific agents in chemotherapy protocols"

Undesirable Effects — How substantial are the undesirable anticipated effects?

JUDGEMENT	RESEARCH EVIDENCE	ADDITIONAL CONSIDERATIONS
Varies	**WAO 2020 Anaphylaxis Guidance** notes that "2nd generation antihistamines may overcome unwanted side effects such as sedation which may be counterproductive in anaphylaxis, but first generation H1-antihistamines are currently the only available for parenteral use (e.g. chlorphenamine). Rapid intravenous administration of 1st generation such as chlorphenamine can also cause hypotension)… there is a "concern that their administration can delay more urgent measures such as repeated administration of parenteral epinephrine."	

Certainty of evidence — What is the overall certainty of the evidence of effects?

JUDGEMENT	RESEARCH EVIDENCE	ADDITIONAL CONSIDERATIONS
Low	**EAACI 2014, WAO Update 2015:** No high quality evidence from RCTs or quasi-RCTs supports the use of antihistamines in the treatment of anaphylaxis: very low certainty evidence to recommend antihistamines. The ASCIA 2020 Guideline recommends against antihistamines for the acute management of anaphylaxis.	

	**JTFPP 2021** suggest against administering antihistamines as an intervention to prevent biphasic anaphylaxis. Conditional recommendation. Certainty rating of evidence: very low. The JTFPP were concerned that "antihistamine administration could lead to a delay in first-line treatment of anaphylaxis". No clear benefit was identified in preventing biphasic reactions with histamine 1 (H1) antihistamines (OR, 0.71; 95% CI, 0.47-1.06) or H2 antihistamines (OR, 1.21; 95% CI, 0.80-1.83). Assuming a 5% rate of biphasic reactions, the number needed to treat (NNT) for H1 antihistamines is 72 to prevent 1 biphasic reaction, with significant uncertainty in the estimate.<br><br>There are significant concerns that more patients presenting with anaphylaxis to Emergency Departments are treated with antihistamines, than receive adrenaline, despite increasing emphasis on adrenaline as the first-line intervention. Wiley & Romo (2020) found an association between antihistamine administration and delayed presentation to hospital in paediatric patients with anaphylaxis. In the European Anaphylaxis Register, antihistamine treatment was significantly associated with the occurrence of biphasic reactions (OR 1.52, 95%CI 1.14-2.02); it has been suggested that this may be due to the use of antihistamines causing a delay in the appropriate use of adrenaline.	

Values

Is there important uncertainty about or variability in how much people value the main outcomes?

JUDGEMENT	RESEARCH EVIDENCE	ADDITIONAL CONSIDERATIONS
Probably no important uncertainty or variability	Reducing symptoms and the risk of a biphasic reaction/return to the Emergency Department are important, both in terms of patient outcomes and cost effectiveness. Biphasic reactions are more likely to be associated with hospital admission.	

Balance of effects

Does the balance between desirable and undesirable effects favour the intervention or the comparison?

JUDGEMENT	RESEARCH EVIDENCE	ADDITIONAL CONSIDERATIONS
Varies	The symptomatic relief of the cutaneous symptoms of anaphylaxis is desirable. However, anecdotal evidence suggests that adrenaline is more effective for cutaneous symptoms than antihistamine, although the effect may be short-lived.<br><br>Thus, antihistamines are favoured provided the potential undesirable effects are mitigated by:<br>• They are not used as part of the emergency treatment of acute anaphylaxis.<br>• They are considered third line after other treatments i.e. adrenaline, oxygen, IV fluids.<br>• Consideration is given to administration via the oral route.<br>• Newer, less sedating antihistamines are given.	

Resources required and cost effectiveness

How large are the resource requirements (costs)? Does the cost-effectiveness of the intervention favour the intervention or the comparison?

JUDGEMENT	RESEARCH EVIDENCE	ADDITIONAL CONSIDERATIONS
Negligible costs and savings	Given the negligible cost of antihistamine treatment and the desirable symptomatic relief they provide, the intervention is probably favoured. The **2020 American Practice Parameter** notes "there would be minimal reduction in costs from omitting treatment with antihistamines."	

Equity, Acceptability, Feasibility

What would be the impact on health equity? Is the intervention acceptable to key stakeholders? Is the intervention feasible to implement?

Equity:<br>probably no impact<br><br>Acceptability:<br>probably yes<br><br>Feasibility: yes	In the context of prioritising life-saving treatments (such as adrenaline) over less effective (or non-effective) interventions, further de-emphasis of antihistamine as a treatment for respiratory/cardiovascular symptoms of acute anaphylaxis is likely to be acceptable to patients (although their use in treating cutaneous features is indicated, once the life-threatening cardiovascular and respiratory features of anaphylaxis have been reversed with adrenaline).	

	JUDGEMENT						
PROBLEM	No	Probably no	Probably yes	**Yes**		Varies	Don't know
DESIRABLE EFFECTS	**Trivial**	Small	Moderate	Large		Varies	Don't know
UNDESIRABLE EFFECTS	Large	Moderate	Small	Trivial		**Varies**	Don't know
CERTAINTY OF EVIDENCE	Very low	**Low**	Moderate	High			No included studies
VALUES	Important uncertainty or variability	Possibly important uncertainty or variability	**Probably no important uncertainty or variability**	No important uncertainty or variability			
BALANCE OF EFFECTS	Favours the comparison	Probably favours the comparison	Favours neither the intervention or comparator	Probably favours the intervention	Favours the intervention	**Varies**	Don't know
RESOURCES REQUIRED	Large costs	Moderate costs	**Negligible costs and savings**	Moderate savings	Large savings	Varies	Don't know
CERTAINTY OF EVIDENCE OF REQUIRED RESOURCES	Very low	Low	Moderate	High			**No included studies**
COST EFFECTIVENESS	Favours the comparison	Probably favours the comparison	**Favours neither the intervention or comparator**	Probably favours the intervention	Favours the intervention	Varies	No included studies
EQUITY	Reduced	Probably reduced	**Probably no impact**	Probably increased	Increased	Varies	Don't know
ACCEPTABILITY	No	Probably no	**Probably yes**	Yes		Varies	Don't know
FEASIBILITY	No	Probably no	Probably yes	**Yes**		Varies	Don't know

CONCLUSIONS

Recommendation

1. **The working group suggests that antihistamines are not used as part of the initial emergency treatment for anaphylaxis (weak recommendation, low certainty evidence).**
   - **antihistamines have no role in treating respiratory or cardiovascular symptoms of anaphylaxis**
2. **Antihistamines can be used to treat skin symptoms which often occur as part of allergic reactions including anaphylaxis (weak recommendation, very low certainty evidence)**
   - **their use must not delay management of respiratory or cardiovascular symptoms of anaphylaxis (using adrenaline and IV fluids)**

Other considerations

- Staff training with emphasis on the need to treat life-threatening features of anaphylaxis prior to cutaneous symptoms which are not life-threatening, whilst acknowledging that adrenaline is very effective against cutaneous symptoms.
- Availability of oral, non-sedating antihistamines in healthcare settings.
- It has been acknowledged that RCTs are required to establish the role of H1- and H2-antihistamines in the treatment of anaphylaxis.

8. Are corticosteroids effective in the treatment of anaphylaxis?

**POPULATION:**	Infants, children, adults and over 65s with suspected anaphylaxis.	**SETTING:**	Hospital and out-of-hospital setting
**INTERVENTION:**	Corticosteroid e.g. hydrocortisone, prednisolone	**COMPARISON:**	No corticosteroid
**MAIN OUTCOMES:**	1. Resolution of symptoms<br>2. Survival with good functional outcome, survival with complications	3. Occurrence of biphasic reaction<br>4. Biphasic reaction which prompts return visit to Emergency Department	

ASSESSMENT

Desirable Effects

How substantial are the desirable anticipated effects?

JUDGEMENT	RESEARCH EVIDENCE	ADDITIONAL CONSIDERATIONS
Varies	The primary action of corticosteroids is the downregulation of late (rather than early phase) inflammatory response. The rationale for the use of corticosteroids is to reduce anaphylaxis severity and/or to prevent biphasic reactions. However, the absorption kinetics and mechanism of action of corticosteroids make it theoretically very unlikely that they are of benefit in the acute treatment of anaphylaxis.<br><br>A Cochrane review (2012) identified no RCTs or quasi RCTs assessing the effectiveness of corticosteroids in the management of anaphylaxis, concluding "clinicians should be aware of the lack of a strong evidence base for the use of a glucocorticoid for anaphylaxis". A 2017 systematic review (which included relevant case series, prospective and retrospective cohort studies, and clinical trials) found no "compelling evidence demonstrating an effective role in reducing anaphylaxis severity or preventing biphasic anaphylaxis." (Alqurashi & Ellis, 2017)<br><br>The **WAO 2020 Anaphylaxis Guidance** notes that "glucocorticosteroids are commonly used in anaphylaxis, with the objective of preventing protracted symptoms, in particular in patients with asthmatic symptoms, and also to prevent biphasic reactions (e.g. intravenous hydrocortisone or methylprednisolone). However, there is increasing evidence that glucocorticosteroids may be of no benefit in the acute management of anaphylaxis, and may even be harmful; their routine use is becoming controversial".<br><br>**The 2020 American Joint Task Force on Practice Parameters (JTFPP)** advises "against glucocorticoids as an intervention to prevent biphasic anaphylaxis" on the basis of very low-quality evidence. The authors reported that at a biphasic anaphylaxis patient expected event rate (PEER) of 5%, the number pf patients needed to treat (NNT) with glucocorticoids is 161 to prevent one episode of biphasic anaphylaxis, with significant uncertainty in the estimate.	**2020 JTFPP** notes that "based on very low-quality evidence, we suggest in favour of the administration of glucocorticoids as an intervention to prevent anaphylaxis in patients undergoing aeroallergen rush immunotherapy … or to prevent anaphylaxis or infusion related reactions when indicated for specific agents in chemotherapy protocols"

Undesirable Effects

How substantial are the undesirable anticipated effects?

JUDGEMENT	RESEARCH EVIDENCE	ADDITIONAL CONSIDERATIONS
Varies	Previous guidelines have commented that it is unlikely that the administration of corticosteroids causes adverse effects in the doses historically recommended for anaphylaxis. Given the lack of evidence for an acute beneficial effect in acute anaphylaxis, the main indication for their use have been for the prevention of biphasic reactions.<br><br>However, as with antihistamine, it has been noted that "glucocorticoids are often inappropriately used as the first-line agent in place of adrenaline, which is more likely to prove life-saving" (Choo et al, 2012). For example, a prospective study in 180 adults and children presenting to the Emergency Department with anaphylaxis identified that only 25% of patients received adrenaline in comparison to 83% who received corticosteroids (Ruiz-Oropeza et al. 2017).<br><br>In a mixed prospective/retrospective cohort of 3498 children and adults presenting to Emergency Departments in Canada, Gabrielli et al (2019) reported an association between prehospital administration of corticosteroids for anaphylaxis and admission to intensive care (adjusted odds ratio 2.8) despite adjusting for reaction severity and other potential confounders. There was no impact on need for adrenaline in the ED. As noted in an accompanying editorial, less than one third of the cohort received timely prehospital epinephrine and one quarter never received it (Campbell, 2019). Campbell concludes "there is a lack of evidence to support the routine use of corticosteroids in the management of anaphylaxis, with the current study adding to the concerns about potential harms, rather than there being simply no benefit".	

**Certainty of evidence** What is the overall certainty of the evidence of effects?		
**JUDGEMENT**	**RESEARCH EVIDENCE**	**ADDITIONAL CONSIDERATIONS**
Very low	**2020 JTFPP** notes "a scarcity of data demonstrating the efficacy of glucocorticoids in the treatment of acute anaphylaxis despite common anecdotal administration in this setting, and no studies have established their benefit when combined with epinephrine and/or antihistamines". **JTFPP 2021** suggest against administering glucocorticoids as an intervention to prevent biphasic anaphylaxis. Conditional recommendation. Certainty rating of evidence: very low. No clear benefit was identified in preventing biphasic reactions with glucocorticoids (OR, 0.87; 95% CI, 0.74-1.02). Assuming a 5% rate of biphasic reactions, the number needed to treat (NNT) for glucocorticoids is 161 to prevent 1 biphasic reaction, with significant uncertainty in the estimate.	

**Values** Is there important uncertainty about or variability in how much people value the main outcomes?		
**JUDGEMENT**	**RESEARCH EVIDENCE**	**ADDITIONAL CONSIDERATIONS**
Probably no important uncertainty or variability	Reducing symptoms and the risk of a biphasic reaction/return to the Emergency Department are important, both in terms of patient outcomes and cost effectiveness. Biphasic reactions are more likely to be associated with hospital admission.	

**Balance of effects** Does the balance between desirable and undesirable effects favour the intervention or the comparison?		
**JUDGEMENT**	**RESEARCH EVIDENCE**	**ADDITIONAL CONSIDERATIONS**
Varies	The historical basis for corticosteroids seems to be that they may be beneficial and do not cause harm. However, more recent data has shown that the assumption over lack of harm is no longer tenable.	

**Resources required and cost effectiveness** How large are the resource requirements (costs)? Does the cost-effectiveness of the intervention favour the intervention or the comparison?		
**JUDGEMENT**	**RESEARCH EVIDENCE**	**ADDITIONAL CONSIDERATIONS**
Varies	Corticosteroids (in the form of hydrocortisone, methylprednisolone and prednisolone) are widely available within hospital settings. Potential for cost savings with reduction in length of hospital stay, but more recent evidence suggests that the opposite may be true. No formal analysis undertaken.	

**Equity, Acceptability, Feasibility** What would be the impact on health equity? Is the intervention acceptable to key stakeholders? Is the intervention feasible to implement?		
**JUDGEMENT**	**RESEARCH EVIDENCE**	**ADDITIONAL CONSIDERATIONS**
Equity:<br>probably no impact<br><br>Acceptability:<br>probably no<br><br>Feasibility: yes	Given the recent concerns over the use of corticosteroids being associated with prolonged hospitalisation and no obvious benefit in decreasing the risk of biphasic reaction, their routine use in anaphylaxis may no longer be acceptable.<br>In the context of prioritising life-saving treatments (such as adrenaline) over less effective (or non-effective) interventions, removal of corticosteroids as a treatment for acute anaphylaxis likely to be acceptable to patients.	

	JUDGEMENT						
PROBLEM	No	Probably no	Probably yes	**Yes**		Varies	Don't know
DESIRABLE EFFECTS	Trivial	Small	Moderate	Large		**Varies**	Don't know
UNDESIRABLE EFFECTS	Large	Moderate	Small	Trivial		**Varies**	Don't know
CERTAINTY OF EVIDENCE	**Very low**	Low	Moderate	High			No included studies
VALUES	Important uncertainty or variability	Possibly important uncertainty or variability	**Probably no important uncertainty or variability**	No important uncertainty or variability			
BALANCE OF EFFECTS	Favours the comparison	Probably favours the comparison	Favours neither the intervention or comparator	Probably favours the intervention	Favours the intervention	**Varies**	Don't know
RESOURCES REQUIRED	Large costs	Moderate costs	Negligible costs and savings	Moderate savings	Large savings	**Varies**	Don't know
CERTAINTY OF EVIDENCE OF REQUIRED RESOURCES	Very low	Low	Moderate	High			**No included studies**
COST EFFECTIVENESS	Favours the comparison	Probably favours the comparison	**Favours neither the intervention or comparator**	Probably favours the intervention	Favours the intervention	Varies	No included studies
EQUITY	Reduced	Probably reduced	**Probably no impact**	Probably increased	Increased	Varies	Don't know
ACCEPTABILITY	No	**Probably no**	Probably yes	Yes		Varies	Don't know
FEASIBILITY	No	Probably no	Probably yes	**Yes**		Varies	Don't know

CONCLUSIONS

Recommendation

1. We suggest *against* the routine use of corticosteroids to treat anaphylaxis (weak recommendation, very low certainty evidence).
There is no strong evidence that corticosteroids are effective in the management of routine anaphylaxis nor the prevention of biphasic reactions, and evidence of low certainty that their use may result in prolonged hospitalization (although it is difficult to fully avoid potential confounding by reverse causation).

The administration of glucocorticoids has been associated with delayed administration of adrenaline, which is the accepted first line and potentially life-saving treatment for anaphylaxis.

2. Corticosteroids may be useful as a third line intervention to treat underlying asthma or shock (weak recommendation, very low certainty evidence)
Corticosteroids may be useful as a 3rd line intervention to treat underlying asthma or shock. Given the altered risk:benefit ratio with respect to severe refractory anaphylaxis, it is reasonable for corticosteroids to be administered as part of the management of refractory reactions, but should not be given in preference to adrenaline or other inotropes/vasopressor agents.

Other considerations

- (Quasi-)RCTs are required to establish the potential benefits and harms of corticosteroids in the treatment of anaphylaxis.

9. Are inhaled beta-2 agonists effective in the treatment of anaphylaxis?

**POPULATION:**	Infants, children, adults and over 65s with suspected anaphylaxis.	**SETTING:**	Hospital and out-of-hospital setting
**INTERVENTION:**	Inhaled bronchodilators e.g. salbutamol	**COMPARISON:**	No inhaled bronchodilator therapy
**MAIN OUTCOMES:**	1. Resolution of symptoms<br>2. Survival with good functional outcome, survival with complications		

ASSESSMENT

JUDGEMENT	RESEARCH EVIDENCE	ADDITIONAL CONSIDERATIONS
**Desirable Effects** — How substantial are the desirable anticipated effects?		
Moderate	There is a lack of evidence of high certainty to support the use of inhaled beta-2 agonists in the emergency treatment of anaphylaxis:<br>• **EAACI 2014 Anaphylaxis guideline** found "no evidence from primary studies for other potential treatments, such as … bronchodilators, and it is therefore not possible to offer any recommendations for the use of these treatments," but then states "inhaled short-acting beta-2 agonists can be given to relieve symptoms of bronchoconstriction in patients with anaphylaxis" on the basis of expert opinion.<br>• **World Allergy Organization anaphylaxis guideline 2011** notes that "extrapolating from their use in acute asthma, selective beta-2 adrenergic agonists such as salbutamol … are sometimes given in anaphylaxis as (2nd line) treatment for wheezing, coughing, and shortness of breath not relieved by epinephrine. Although this is helpful for lower respiratory tract symptoms, these medications should not be substituted for epinephrine because they have minimal alpha-1 adrenergic agonist vasoconstrictor effects and do not prevent or relieve laryngeal oedema and upper airway obstruction, hypotension, or shock"<br>• **ASCIA 2020 Guideline for acute management of anaphylaxis** includes the option to administer beta-2 agonists for persisting wheeze but cautions that "bronchodilators must not be used as first line medication for anaphylaxis as they do not prevent or relieve upper airway obstruction, hypotension or shock."	In the presence of severe bronchoconstriction, the administration of inhaled or nebulised beta-agonists is unlikely to be effective (as with asthma).<br><br>UK and AUSTRALIAN guidelines recommend to administer IM adrenaline FIRST (and then asthma reliever) in someone with known asthma and allergy to food, insect stings or medication who develops sudden breathing difficulty (including wheeze, persistent cough or hoarse voice), even if no skin symptoms are present.
**Undesirable Effects** — How substantial are the undesirable anticipated effects?		
Small	• The use of beta-2 agonists could delay administration of IM adrenaline (both initial and subsequent doses).<br>• B2-agonists have common undesirable side effects such as arrhythmia, dizziness, headache, tremor but these can also occur due to the anaphylaxis reaction and its treatment. Hypokalaemia is common with their use.<br><br>The **EAACI 2014 Anaphylaxis guideline** advises that while "intramuscular adrenaline is first-line treatment in the emergency setting, in controlled circumstances in hospital with clinical staff experienced in managing anaphylaxis (e.g. oral food challenge in an allergy clinic), mild wheeze may initially be treated with inhaled short-acting beta-2 agonists alone; intramuscular adrenaline should be given if there is no response within 5 minutes" on the basis of expert opinion. This strategy could be perceived to imply that the use of IM adrenaline as first-line treatment for anaphylaxis is to be avoided. (Burrell et al. Arch Dis Child. 2020).	
**Certainty of evidence** — What is the overall certainty of the evidence of effects?		
Very low	• **EAACI 2014 Anaphylaxis guideline** found "no evidence from primary studies for other potential treatments, such as … bronchodilators, and it is therefore not possible to offer any recommendations for the use of these treatments".<br>• **World Allergy Organization anaphylaxis guideline 2011** notes that limited evidence is based on "extrapolating from their use in acute asthma"	
**Values** — Is there important uncertainty about or variability in how much people value the main outcomes?		
No important uncertainty or variability	The main outcome is symptom resolution and survival with good functional outcome – this is universally highly valued.	

Balance of effects

Does the balance between desirable and undesirable effects favour the intervention or the comparison?

JUDGEMENT	RESEARCH EVIDENCE	ADDITIONAL CONSIDERATIONS
Favours the intervention	The relief of lower respiratory tract symptoms / bronchospasm is desirable and if severe / with concurrent asthmatic overlay, can be life-saving.	Beta-2 agonists must not be used in preference to adrenaline.

Resources required and cost effectiveness

How large are the resource requirements (costs)? Does the cost-effectiveness of the intervention favour the intervention or the comparison?

JUDGEMENT	RESEARCH EVIDENCE	ADDITIONAL CONSIDERATIONS
Negligible costs and savings	B2-agonists are inexpensive and widely available both in hospital and the community. No formal analysis undertaken, but probably cost-effective.	

Equity, Acceptability, Feasibility

What would be the impact on health equity? Is the intervention acceptable to key stakeholders? Is the intervention feasible to implement?

JUDGEMENT	RESEARCH EVIDENCE	ADDITIONAL CONSIDERATIONS
Equity:<br>probably no impact<br><br>Acceptability: yes<br><br>Feasibility: yes	Beta-2 agonists are already widely used in clinical practice and in most anaphylaxis guidelines as a 2nd line treatment.<br>They are accepted as a treatment for lower respiratory symptoms both in hospital settings and the community.	

	JUDGEMENT						
**PROBLEM**	No	Probably no	Probably yes	**Yes**		Varies	Don't know
**DESIRABLE EFFECTS**	Trivial	Small	**Moderate**	Large		Varies	Don't know
**UNDESIRABLE EFFECTS**	Large	Moderate	**Small**	Trivial		Varies	Don't know
**CERTAINTY OF EVIDENCE**	**Very low**	Low	Moderate	High			No included studies
**VALUES**	Important uncertainty or variability	Possibly important uncertainty or variability	Probably no important uncertainty or variability	**No important uncertainty or variability**			
**BALANCE OF EFFECTS**	Favours the comparison	Probably favours the comparison	Favours neither the intervention or comparator	Probably favours the intervention	**Favours the intervention**	Varies	Don't know
**RESOURCES REQUIRED**	Large costs	Moderate costs	**Negligible costs and savings**	Moderate savings	Large savings	Varies	Don't know
**CERTAINTY OF EVIDENCE OF REQUIRED RESOURCES**	Very low	Low	Moderate	High			**No included studies**
**COST EFFECTIVENESS**	Favours the comparison	Probably favours the comparison	**Favours neither the intervention or comparator**	Probably favours the intervention	Favours the intervention	Varies	No included studies
**EQUITY**	Reduced	Probably reduced	**Probably no impact**	Probably increased	Increased	Varies	Don't know
**ACCEPTABILITY**	No	Probably no	Probably yes	**Yes**		Varies	Don't know
**FEASIBILITY**	No	Probably no	Probably yes	**Yes**		Varies	Don't know

CONCLUSIONS

Recommendation

1. **Beta-2 agonists (such as salbutamol) may be useful as an adjunct treatment for lower respiratory symptoms caused by anaphylaxis, following initial treatment with IM adrenaline (weak recommendation, very low certainty evidence).**
2. **In the presence of persisting respiratory symptoms in anaphylaxis, beta-2 agonists (whether inhaled or parenteral) should not be used as an alternative to further parenteral treatment with adrenaline (strong recommendation, very low certainty evidence).**

The first line treatment for respiratory symptoms in the context of anaphylaxis is IM adrenaline. We recommend the use of B2-agonists as an adjunctive / 2nd-line treatment in patients with the lower respiratory tract symptoms of bronchoconstriction (cough, wheeze, poor air entry, shortness of breath). Beta-2 agonists must not be used as an alternative to further doses of adrenaline where indicated.

In patients with mild to moderate respiratory symptoms, beta-2 agonists can be administered by repeated activations of a pMDI via an appropriate large volume spacer. There are insufficient data on which to make a recommendation about the use of metered dose inhalers with spacers in acute-severe or life-threatening respiratory symptoms; in these patients, beta-agonists should be administered by an oxygen-driven nebuliser.

In the presence of refractory anaphylaxis, adrenaline infusion should be used as a preferred intervention for management (and not intravenous infusion of salbutamol).

Other considerations

- RCTs unlikely for ethical reasons given the knowledge that inhaled B2-agonists are effective at relieving bronchoconstriction, knowledge extrapolated from asthma treatment.
- There is no evidence base (for or against) the use of intravenous beta-2 agonists such as salbutamol in the management of anaphylaxis.

UK and AUSTRALIAN guidelines recommend to administer IM adrenaline FIRST (and then asthma reliever) in someone with known asthma and allergy to food, insect sting or medication who develops sudden breathing difficulty (including wheeze, persistent cough or hoarse voice) even if no skin symptoms are present.

Caution is recommended if using Intravenous magnesium sulphate (as an alternative to beta-2 agonists), as this can cause significant vasodilation and worsen hypotension in the context of anaphylaxis.

10. How long should patients be observed in hospital following anaphylaxis?

**POPULATION:**	Infants, children, adults and over 65s with suspected anaphylaxis.	**SETTING:**	Hospital and out-of-hospital setting
**INTERVENTION:**	"Prolonged" observation in hospital following suspected anaphylaxis	**COMPARISON:**	Discharge following resolution of symptoms of anaphylaxis
**MAIN OUTCOMES:**	1. Occurrence of biphasic reaction<br>2. Biphasic reaction which prompts return visit to Emergency Department		3. Death due to biphasic reaction<br>4. Survival with good functional outcome, survival with complications

ASSESSMENT

Desirable Effects

How substantial are the desirable anticipated effects?

JUDGEMENT	RESEARCH EVIDENCE	ADDITIONAL CONSIDERATIONS
Moderate	Anaphylaxis may resolve but then exhibit a recrudescence several hours later in the absence of further exposure to allergen. This is termed 'biphasic anaphylaxis', but can also represent (and be difficult to distinguish from) protracted anaphylaxis with a transient response to adrenaline, or in the case of food-induced reactions, further allergen absorption from the gastrointestinal tract.<br><br>Previous guidelines have estimated a rate of up to 20% for biphasic reactions, however a recent meta-analysis reported a pooled rate of 4.6% (95%CI 4.0, 5.3) (Lee et al, 2015). A rate of 4.7% has been reported by the European Anaphylaxis Registry in a cohort of over 9000 reactions (Kraft et al, 2020). In a prospective case series of anaphylaxis presenting to Australian Emergency Departments, delayed deteriorations were noted in 17% (55/315) reactions, of which 29 (9.2%) required treatment with adrenaline (Brown et al, 2014).<br><br>Contradictory ranges for the onset of biphasic symptoms are reported in the literature:<br>• **WAO anaphylaxis guideline 2011** states "symptoms recur within 1–72 hours (usually within 8 –10 hours) after the initial symptoms have resolved."<br>• **EAACI 2014 Anaphylaxis guideline** state symptoms "usually occur within 4-12 hours of the first symptoms"<br>• Brown et al (2014) reported a median onset for delayed deteriorations of 1.7 (IQR 0.7-4.3) hours after arrival to the ED. Importantly, all patients requiring adrenaline either had severe initial presentation or had incomplete resolution of initial symptoms prior to deterioration.<br>• A recent systematic review and meta-analysis of 27 studies reported that the mean and median time from initial reaction to onset of biphasic symptoms was 10 and 11 hours, respectively i.e. 50% of biphasic reactions begin after 11 hours from initial symptoms (Lee et al, 2015).<br><br>The optimal duration of observation following anaphylaxis (to monitor for biphasic reactions) is unknown:<br>• A recent meta-analysis of 12 studies (2890 adults with anaphylaxis, 4.9% with biphasic reaction) suggested that a 1 hour observation would capture 95.0% (95%CI 99.0%, 97.3%) of biphasic reactions. Observing patients for four, six and twelve hours would capture 96.5% (95%CI 93.4%, 98.2%), 97.3% (95%CI 95.0%, 98.5%) and 98.2% (95%CI 96.7%, 99.1%) of biphasic reactions, respectively (Kim et al, 2019).<br>• In an analysis of data from the UK Fatal Anaphylaxis Register, death never occurred more than six hours after contact with the trigger (Pumphrey, 2000); this formed the basis of the previous RCUK recommendation that patients should be observed for at least 6 hours. However, in an updated analysis, 2.5% of fatalities experienced cardiorespiratory arrest more than 6 hours after allergen exposure; 8% and 2.9% occurred after 2 and 4 hours, respectively (Pumphrey & Sturm, 2014).<br><br>**NICE** (2011) concluded there was "no evidence on the effectiveness of observing people... or how long people should be observed after a suspected anaphylactic reaction", but (on the basis of expert opinion) recommended 6–12 hours observation from the onset of symptoms.<br><br>**The ASCIA 2020 Guideline for acute management of anaphylaxis** recommends observation for at least 4 hours after the last dose of adrenaline, and overnight observation if any of the following are relevant: anaphylaxis was severe or protracted anaphylaxis (e.g. required repeated doses of adrenaline or IV fluid resuscitation); the patient has a history of severe/protracted anaphylaxis; concomitant illness (e.g. severe asthma, history of arrhythmia, systemic mastocytosis); patient lives alone or is remote from medical care; patient presents for medical care late at night.<br><br>**The 2020 JTFPP** undertook a systematic review and meta-analysis of 32 studies, and found that "biphasic anaphylaxis is associated with a more severe initial presentation of anaphylaxis (OR=2.11, 95% CI 1.23-3.61) or >1 dose of adrenaline required with the initial presentation (OR 4.82, 95% CI 2.70-8.58)." On the basis of these data, they concluded that "very low-quality evidence suggests extended observation is appropriate for patients with [more] severe initial anaphylaxis. For patients with resolved non-severe anaphylaxis who are without significant co-morbidities that would increase the risk for fatal anaphylaxis, who have had a prompt response to epinephrine, and will have reliable access to medical care following discharge, a 1-hour observation may be reasonable." Some studies have reported a delay in adrenaline administration (of >30-60 minutes from symptom onset) as being associated with an increased risk of biphasic reactions (Liu et al, 2020) but the data are not consistent and could not be included in meta-analysis.	**NICE (2011)** recommends that children (under 16 years) should be admitted to hospital under the care of a paediatric medical team. This is because "for many children and their parents and/or carers a suspected anaphylactic reaction is a traumatic experience and will raise many different issues. It is important for children and their parents or carers to receive the appropriate care (for example, paediatric assessment, counselling, education) following emergency treatment. Therefore all children should be admitted to hospital following emergency treatment, to be cared for by a paediatric medical team."<br><br>NICE further acknowledges that "shorter observation periods could be warranted in those who seek and respond quickly to treatment."

**Undesirable Effects** How substantial are the undesirable anticipated effects?		
**JUDGEMENT**	**RESEARCH EVIDENCE**	**ADDITIONAL CONSIDERATIONS**
Varies	**2020 JTFPP** notes that "the majority of patients monitored for 1 asymptomatic hour after resolved anaphylaxis will not experience a biphasic reaction. Therefore the risks and benefits need to be balanced. While harm may result from missed cases of anaphylaxis in discharged patients, an overly cautious observation time for patients at low risk for both biphasic anaphylaxis and anaphylaxis fatality would be very costly… undesirable effects could include prolonged periods of medical observation which would be unnecessary for the majority of patients with resolved anaphylaxis."	**2020 JTFPP** further notes that some patients "may be reluctant to go the Emergency Department for fear of having an extended stay."

**Certainty of evidence** What is the overall certainty of the evidence of effects?		
**JUDGEMENT**	**RESEARCH EVIDENCE**	**ADDITIONAL CONSIDERATIONS**
Very low	There is very low certainty over the specific risk factors for biphasic reactions. However, in terms of preventing fatalities, data from the UK Fatal Anaphylaxis Registry (over 20 years of data) indicate that i) fatal anaphylaxis is a very rare outcome, and ii) 90% and 95% of fatalities occur within 2 and 4 hours of allergen exposure, respectively.	

**Values** Is there important uncertainty about or variability in how much people value the main outcomes?		
**JUDGEMENT**	**RESEARCH EVIDENCE**	**ADDITIONAL CONSIDERATIONS**
Possibly important uncertainty or variability	While patients would clear prefer to avoid biphasic reactions, and if they did occur would want to be in a safe environment, delayed fatal reactions are so rare that it is likely there is a trade-off between prolonged observation and patient/carer wishes in terms of discharge preference.	

**Balance of effects** Does the balance between desirable and undesirable effects favour the intervention or the comparison?		
**JUDGEMENT**	**RESEARCH EVIDENCE**	**ADDITIONAL CONSIDERATIONS**
Probably favours the intervention	Although prolonged observation may be inconvenient, patients (and their families) are more likely to be concerned over risk to life.	

**Resources required and cost effectiveness** How large are the resource requirements (costs)? Does the cost-effectiveness of the intervention favour the intervention or the comparison?		
**JUDGEMENT**	**RESEARCH EVIDENCE**	**ADDITIONAL CONSIDERATIONS**
Moderate costs	Cost-effectiveness favours comparison due to the rarity of fatal anaphylaxis beyond 2-4 hours after initial allergen exposure. NICE acknowledges that "because the recommendation [for 6-12 hours observation] did not represent a major departure from current practice, the likely cost impact could be assumed to be relatively small. [However] health economic modelling could potentially provide a useful exploration of the trade-offs between cost, benefit and safety in this area."<br>Cost-effective modelling of prolonged observation (>6 hours) has been undertaken in the US context, and concluded that "prolonged medical observation may not be cost-effective for patients at low risk for biphasic anaphylaxis; however, where costs are low (<$46/hr) or risk of biphasic reaction high (>17%), longer medical observation can be justified." (Shaker et al, 2019)	NICE notes "in patients with reactions that are controlled promptly and easily, a shorter observation period may be considered provided that they receive appropriate post-reaction care prior to discharge."

**Equity, Acceptability, Feasibility** What would be the impact on health equity? Is the intervention acceptable to key stakeholders? Is the intervention feasible to implement?		
Equity: probably increased<br>Acceptability: varies<br>Feasibility: yes	Ensure all patients have equal access to best anaphylaxis care in accordance with the NHS constitution. Prolonged observation is inconvenient for many patients and their carers.<br>Prolonged observation (typically 6-12 hours) is currently recommended by NICE, although the NICE guideline acknowledges that this is on the basis of expert opinion as "No evidence on the effectiveness of observing people after a suspected anaphylactic reaction was identified. No evidence on for how long people should be observed after a suspected anaphylactic reaction was identified."	

	JUDGEMENT						
**PROBLEM**	No	Probably no	Probably yes	**Yes**		Varies	Don't know
**DESIRABLE EFFECTS**	Trivial	Small	**Moderate**	Large		Varies	Don't know
**UNDESIRABLE EFFECTS**	Large	Moderate	Small	Trivial		**Varies**	Don't know
**CERTAINTY OF EVIDENCE**	**Very low**	Low	Moderate	High			No included studies
**VALUES**	Important uncertainty or variability	**Possibly important uncertainty or variability**	Probably no important uncertainty or variability	No important uncertainty or variability			
**BALANCE OF EFFECTS**	Favours the comparison	Probably favours the comparison	Favours neither the intervention or comparator	**Probably favours the intervention**	Favours the intervention	Varies	Don't know
**RESOURCES REQUIRED**	Large costs	**Moderate costs**	Negligible costs and savings	Moderate savings	Large savings	Varies	Don't know
**CERTAINTY OF EVIDENCE OF REQUIRED RESOURCES**	Very low	Low	**Moderate**	High			No included studies
**COST EFFECTIVENESS**	Favours the comparison	**Probably favours the comparison**	Favours neither the intervention or comparator	Probably favours the intervention	Favours the intervention	Varies	No included studies
**EQUITY**	Reduced	Probably reduced	Probably no impact	**Probably increased**	Increased	Varies	Don't know
**ACCEPTABILITY**	No	Probably no	Probably yes	Yes		**Varies**	Don't know
**FEASIBILITY**	No	Probably no	Probably yes	**Yes**		Varies	Don't know

CONCLUSIONS

Recommendation

A risk-stratified approach to discharge is suggested, as shown in Table 3 (weak recommendation):

**Consider fast-track discharge (after 2 hours observation) if:**	**Minimum 6 hours observation after resolution of symptoms recommended if:**	**Observation for at least 12 hours following resolution of symptoms if any one of the following:**
• Good response to a single dose of adrenaline given within 30 minutes of symptom onset;<br>AND<br>• Complete resolution of symptoms;<br>AND<br>• The patient already has adrenaline auto-injectors (AAI) and has been trained how to use them.<br>AND<br>• There is adequate supervision following discharge	• 2 doses of IM adrenaline needed to treat reaction*<br>OR<br>• Previous biphasic reaction<br><br>*it may be reasonable for such patients to be discharged after 2 hours e.g. on the advice of an allergy specialist.	• Severe reaction requiring >2 doses of adrenaline.<br>• Patient has severe asthma or reaction involved severe respiratory compromise.<br>• Possibility of continuing absorption of allergen e.g. slow release medicines.<br>• Patient presents late at night, or may not be able to respond to any deterioration.<br>• Patients in areas where access to emergency care is difficult.
